# Effects and potential mechanisms of astragaloside IV in animal models of renal fibrosis: a systematic review and meta-analysis

**DOI:** 10.3389/fphar.2026.1867891

**Published:** 2026-08-25

**Authors:** Shijie Li, Shiyu Zhang, Lin Cui, Yue Li, Yuqi Wang, Youping Wang

**Affiliations:** 1 Division of Cardiology, First Affiliated Hospital, Henan University of Traditional Chinese Medicine, Zhengzhou, China; 2 Collaborative Innovation Center of Prevention and Treatment of Major Diseases by Chinese and Western Medicine, Zhengzhou, China

**Keywords:** astragaloside IV, inflammation, meta-analysis, oxidative stress, renal fibrosis, systematic review

## Abstract

**Background:**

Renal fibrosis is the principal pathological hallmark of progressive chronic kidney disease (CKD). However, the clinical availability of effective, targeted anti-fibrotic therapeutics remains severely constrained. Astragaloside IV (AS-IV), a predominant bioactive metabolite isolated from Astragali Radix, has exhibited substantial renoprotective and anti-fibrotic properties in diverse preclinical models.

**Materials and Methods:**

We systematically searched eight electronic databases (PubMed, Web of Science, Embase, ProQuest, CNKI, SinoMed, VIP, and Wanfang Data) from inception to 30 November 2025, to identify relevant *in vivo* studies. Risk of bias was assessed using the SYRCLE tool. Statistical analyses were performed using Review Manager 5.3 and Stata 18.0.

**Results:**

A total of 34 studies involving 769 animals were included. Compared with the control group, AS-IV treatment significantly reduced the levels of serum creatinine (Scr), blood urea nitrogen (BUN), proteinuria, transforming growth factor-β1 (TGF-β1), fibronectin, and α-smooth muscle actin (α-SMA) (all *P* < 0.0001). Subgroup analyses revealed that intragastric dose was a source of heterogeneity for Scr; publication year for BUN; treatment duration for TGF-β1; and the underlying animal disease model alongside treatment duration for α-SMA. Dose-response analyses revealed that Scr, BUN, TGF-β1, fibronectin, and α-SMA showed overall negative dose-dependent trends. In addition, AS-IV significantly modulated the expression of other indicators related to fibrosis, oxidative stress, and inflammatory responses. Methodologically, the overall quality of the included studies was suboptimal, and there was considerable statistical heterogeneity. Additionally, the presence of publication bias cannot be definitively excluded. These results suggest that AS-IV holds renoprotective potential in animal models of renal fibrosis; however, these results should be interpreted prudently.

**Conclusion:**

This study provides the first systematic review evaluating AS-IV across etiologically diverse models of renal fibrosis, showing its broad-spectrum anti-fibrotic pharmacological effects. The synthesized evidence suggests that these renoprotective effects may be mediated through multi-target modulation, including mitigating inflammation and oxidative stress, modulating autophagy and apoptosis, and reducing extracellular matrix (ECM) accumulation. Nevertheless, given the significant heterogeneity and overall suboptimal methodological quality of the included studies, these positive findings should be interpreted with caution.

**Systematic Review Registration:**

https://www.crd.york.ac.uk/PROSPERO/view/CRD420261372263, identifier CRD420261372263.

## 1 Introduction

The prevalence of chronic kidney disease (CKD) continues to rise, establishing CKD as a leading cause of global mortality (Francis et al., 2024). As a progressive condition, CKD often culminates in kidney failure, a stage where survival relies heavily on costly renal replacement therapy (RRT), such as dialysis or transplantation. However, RRT does not offer a definitive cure, and due to socioeconomic disparities, these life-saving interventions remain inaccessible to many patients globally (Anand et al., 2013). Consequently, identifying effective strategies to delay CKD progression has become a pressing clinical imperative.

Renal fibrosis is the common pathological hallmark and final pathway of CKD, driving the progressive loss of renal function toward end-stage kidney disease (ESKD). Persistent renal injury from diverse etiologies creates a profibrotic microenvironment, triggering the excessive accumulation of extracellular matrix (ECM) components. This deposition disrupts renal architecture and impairs renal function, ultimately driving irreversible organ damage (Humphreys, 2018). Given the prominent role of the transforming growth factor-beta (TGF-β) family in this pathogenic process, the development of specific inhibitors targeting the TGF-β signaling pathway has been a focal point in nephrology research for over 3 decades (Meng et al., 2016). Despite promising preclinical outcomes, clinical trials have often yielded disappointing results. In addition to demonstrating limited therapeutic efficacy, direct systemic blockade often precipitates unacceptable adverse effects (Voelker et al., 2017; Vincenti et al., 2017). This is because TGF-β plays indispensable physiological roles in tissue homeostasis and immune regulation (Deng et al., 2024; Jing et al., 2025). In addition, clinical trials investigating single small-molecule therapies targeting specific inflammatory or chemotactic pathways have reinforced that relying on a single therapeutic target is insufficient, given the vast and complex molecular mechanisms driving fibrosis (Perez-Gomez et al., 2015). Therefore, contemporary anti-fibrotic research has pivoted toward holistic interventions and multi-target therapeutic strategies (Li et al., 2022). To circumvent the severe toxicity associated with direct systemic TGF-β blockade, ideal therapeutic candidates must be pleiotropic agents capable of holistically modulating the fibrotic microenvironment and its downstream cascades without completely abolishing basal, physiological TGF-β signaling.

Botanical therapeutics derived from Traditional Chinese Medicine are increasingly recognized by the global biomedical community for their potential in achieving sophisticated multi-target pharmacological modulation. Retrospective clinical cohorts and real-world evidence suggest that the integrated application of these phytomedicines can significantly enhance the long-term survival trajectories of patients with CKD, with Astragali Radix (commonly known as Huangqi) frequently serving as a cornerstone within these therapeutic regimens (Huang et al., 2018). As the dried root of *Astragalus mongholicus Bunge* [Fabaceae; Astragali Radix], Astragali Radix is widely used in East and Southeast Asian medicine. According to the Pharmacopoeia of the People’s Republic of China, its traditional clinical indications include tonifying *Qi*, raising *Yang*, inducing diuresis, and alleviating edema. These traditional uses have historically underpinned its application in renal therapeutics. Modern pharmacological research confirms that Astragali Radix exhibits considerable renoprotective properties, including the reduction of proteinuria and the suppression of inflammation, oxidative stress, renal fibrosis, and cellular apoptosis (Shi et al., 2024).

Astragaloside IV (AS-IV) is a cycloartane-type tetracyclic triterpenoid saponin extracted from Astragali Radix (CAS number: 84687-43-4), with the molecular formula C_41_H_68_O_14_ and a molecular weight of 784.97. As the most extensively studied active metabolite among the over 200 metabolites identified in Astragali Radix, it has been shown to possess potent anti-inflammatory, anti-oxidative, and anti-fibrotic properties, alongside its ability to improve mitochondrial function and confer cardioprotective and neuroprotective effects (Liu et al., 2024; Yang et al., 2022). The therapeutic effects of AS-IV in cardiac, pulmonary, and hepatic fibrosis have been systematically reviewed (Zhang et al., 2025a; Zhang et al., 2025b; Dan et al., 2024). Although numerous studies have confirmed its effects in treating CKD—as exemplified by a systematic review evaluating AS-IV in diabetic kidney disease (DKD) models—a thorough analysis assessing its pleiotropic anti-fibrotic effects across etiologically diverse renal models remains lacking (Zhou et al., 2020). Therefore, to explore therapeutic candidates that circumvent the translational bottlenecks of systemic TGF-β inhibitors, this study systematically reviews the evidence that AS-IV attenuates renal fibrosis through alternative downstream targets and multi-pathway modulation.

Preclinical systematic reviews are essential for minimizing unnecessary research duplication, optimizing *in vivo* experimental designs, and providing robust evidence-based support for novel drug development and clinical translation. To this end, this study provides a comprehensive synthesis of all available *in vivo* preclinical evidence regarding the application of AS-IV across etiologically diverse animal models of renal fibrosis. The primary objective of this systematic review and meta-analysis is to quantify the renoprotective effects of AS-IV, critically appraise the methodological quality of the existing literature, and systematically summarize the reported multi-target pharmacological mechanisms—particularly its role in mitigating inflammation and oxidative stress, modulating autophagy and apoptosis, and reducing ECM accumulation. By doing so, we aim to establish a strong, evidence-based foundation to guide future standardized preclinical designs and accelerate the clinical translation of AS-IV as an anti-fibrotic therapeutic.

## 2 Materials and methods

This systematic review was conducted in accordance with the Preferred Reporting Items for Systematic Reviews and Meta-Analyses (PRISMA) guidelines (Page et al., 2021). The protocol was registered in the PROSPERO database (CRD420261372263).

### 2.1 Search strategy

Two independent reviewers (Shijie Li and Shiyu Zhang) conducted a comprehensive literature search across eight electronic databases to identify studies investigating the effects of AS-IV on renal fibrosis. The databases searched included PubMed, Web of Science (WoS), Embase, ProQuest, China National Knowledge Infrastructure (CNKI), SinoMed, VIP, and Wanfang Data. The search was restricted to articles published in English and Chinese, encompassing records from the inception of each database up to 30 November 2025. We combined controlled vocabulary (medical subject headings terms and Chinese Medical Subject Headings) with free-text search terms to capture concepts related to the target disease and intervention, ensuring broad coverage of the relevant literature. The detailed search strategy employed for PubMed is provided in Table 1.

**TABLE 1:** Literature search strategy for AS-IV in the treatment of renal fibrosis.

No.	Search items
1	Kidney fibrosis [title/abstract]
2	Renal fibrosis [title/abstract]
3	Nephrofibrosis [title/abstract]
4	Glomerulosclerosis [title/abstract]
5	Tubulointerstitial fibrosis [title/abstract]
6	UUO [title/abstract]
7	Unilateral ureteral obstruction [title/abstract]
8	1 OR 2 OR 3 OR 4 OR 5 OR 6 OR 7
9	Astragaloside IV [mesh]
10	8 and 9

### 2.2 Inclusion and exclusion criteria

The prespecified inclusion criteria, structured according to the PICO framework, were as follows: (1) Population: animal models of renal fibrosis; (2) Intervention: monotherapy with AS-IV at any dosage; (3) Comparator: an equivalent volume of vehicle control (e.g., normal saline or carboxymethyl cellulose) or an untreated disease model cohort; and (4) Outcomes: to ensure a thorough evaluation of renal function and fibrotic progression, eligible studies were required to report at least one of the following primary outcomes: serum creatinine (Scr), blood urea nitrogen (BUN), proteinuria, TGF-β1, fibronectin, or alpha-smooth muscle actin (α-SMA). Secondary outcomes included renal histopathology, urinary albumin-to-creatinine ratio (UACR), type I and IV collagen, E-cadherin, Smad7, phosphorylated Smad3 (p-Smad3), malondialdehyde (MDA), heme oxygenase-1 (HO-1), tumor necrosis factor-alpha (TNF-α), interleukin-1β (IL-1β), interleukin-6 (IL-6), and nuclear factor kappa B (NF-κB). The prespecified exclusion criteria were as follows: (1) *in vitro* studies, clinical trials, and review articles; (2) studies utilizing non-renal fibrosis models; (3) conference abstracts, editorials, and unpublished dissertations; (4) duplicate publications; and (5) studies failing to report any of the predefined primary outcomes. A summary of primary outcomes is shown in Supplementary Table S1.

### 2.3 Data extraction

Two independent reviewers (Shijie Li and Yue Li) screened titles and abstracts, followed by full-text evaluations, to identify eligible studies based on the predefined inclusion and exclusion criteria. Any discrepancies regarding study selection were resolved through consensus or consultation with a third reviewer (Youping Wang). Data were extracted using a standardized Microsoft Excel template, capturing the following variables: (1) first author and publication year; (2) animal characteristics (e.g., species, strain, sex, and body weight); (3) methods of renal fibrosis induction; (4) intervention protocols (including dosage, route of administration, and duration); and (5) primary and secondary outcomes. For studies reporting outcomes at multiple time points, only data from the final time point prior to euthanasia were extracted. If data were presented exclusively in graphical format, digital quantification software (ImageJ version 1.53k) was utilized to extract the numerical values. For studies involving multiple dose regimens, data from each dose level were collected and distinguished by appending H (high), M (middle), and L (low) after the study identifier.

### 2.4 Risk of bias assessment

Two independent reviewers (Shijie Li and Shiyu Zhang) assessed the risk of bias in the included studies using the Systematic Review Centre for Laboratory Animal Experimentation (SYRCLE) risk of bias tool. This instrument evaluates methodological quality across ten specific domains: (1) sequence generation; (2) baseline characteristics; (3) allocation concealment; (4) random housing; (5) blinding of caregivers and investigators; (6) random outcome assessment; (7) blinding of outcome assessors; (8) incomplete outcome data; (9) selective outcome reporting; and (10) other sources of bias. Each domain was independently graded as indicating a “low,” “high,” or “unclear” risk of bias. Any discrepancies between the reviewers were resolved through consensus or consultation with a third reviewer (Youping Wang).

### 2.5 Statistical analysis

Statistical analyses were performed using Review Manager version 5.3 and Stata version 18.0. For continuous outcomes, effect estimates were calculated as standardized mean differences (SMDs) with 95% confidence intervals (CIs). Given inherent methodological and biological variations across *in vivo* studies, a random-effects model was used to pool the data. Statistical significance was defined as *P* < 0.05. Statistical heterogeneity among the included studies was evaluated using the Chi-square (χ^2^) test and the *I* ^*2*^ statistic. For studies involving multiple dose regimens, the sample size (N) of the shared control group was distributed as evenly as possible among the corresponding experimental groups to avoid unit-of-analysis errors caused by repeated counting of the control group. For outcomes exhibiting considerable heterogeneity (defined as *I* ^*2*^ > 50%), sensitivity analyses were conducted using a leave-one-out method. This involved sequentially omitting individual studies and recalculating the pooled effect sizes to determine whether any single study disproportionately influenced the overall results.

### 2.6 Subgroup analysis

To explore potential sources of heterogeneity, subgroup analyses were conducted for the primary outcomes: Scr, BUN, proteinuria, TGF-β1, fibronectin, and α-SMA. The data were stratified according to publication year (pre-2020 vs. 2020 and later), underlying animal disease model (DKD, unilateral ureteral obstruction [UUO], and ischemia-reperfusion injury [IRI]), intragastric dose (≤30 mg/kg, > 30 and ≤ 60 mg/kg, and > 60 mg/kg), and treatment duration (<6 weeks vs. ≥ 6 weeks). These covariates were selected because they were the most frequently and consistently reported variables across the included studies. Differences between subgroups were evaluated using Cochran’s Q test, with *P* < 0.05 considered to indicate a statistically significant subgroup effect.

### 2.7 Publication bias

Funnel plots were generated using RevMan 5.3 to visually evaluate potential publication bias based on plot symmetry. Additionally, Egger’s test (with *P* < 0.10 considered statistically significant) and the non-parametric trim-and-fill method were performed using Stata 18.0 to quantitatively assess this bias and verify the robustness of the pooled estimates.

### 2.8 Dose-response analysis

A dose-response meta-analysis was conducted to quantitatively evaluate the continuous association between escalating doses and the pooled SMDs. To rigorously assess potential non-linear dose-response relationships, a restricted cubic spline model was initially fitted, with three knots located at the 10th, 50th, and 90th percentiles of the non-zero dose distribution. A Wald test was used to examine the statistical significance of the non-linear term. According to the principle of parsimony, if the non-linear trend was not statistically significant (*P* > 0.05), the model was reverted to a standard linear dose-response analysis. All dose-response analyses were performed using Stata 18.0.

## 3 Results

### 3.1 Study inclusion

**FIGURE 1:**
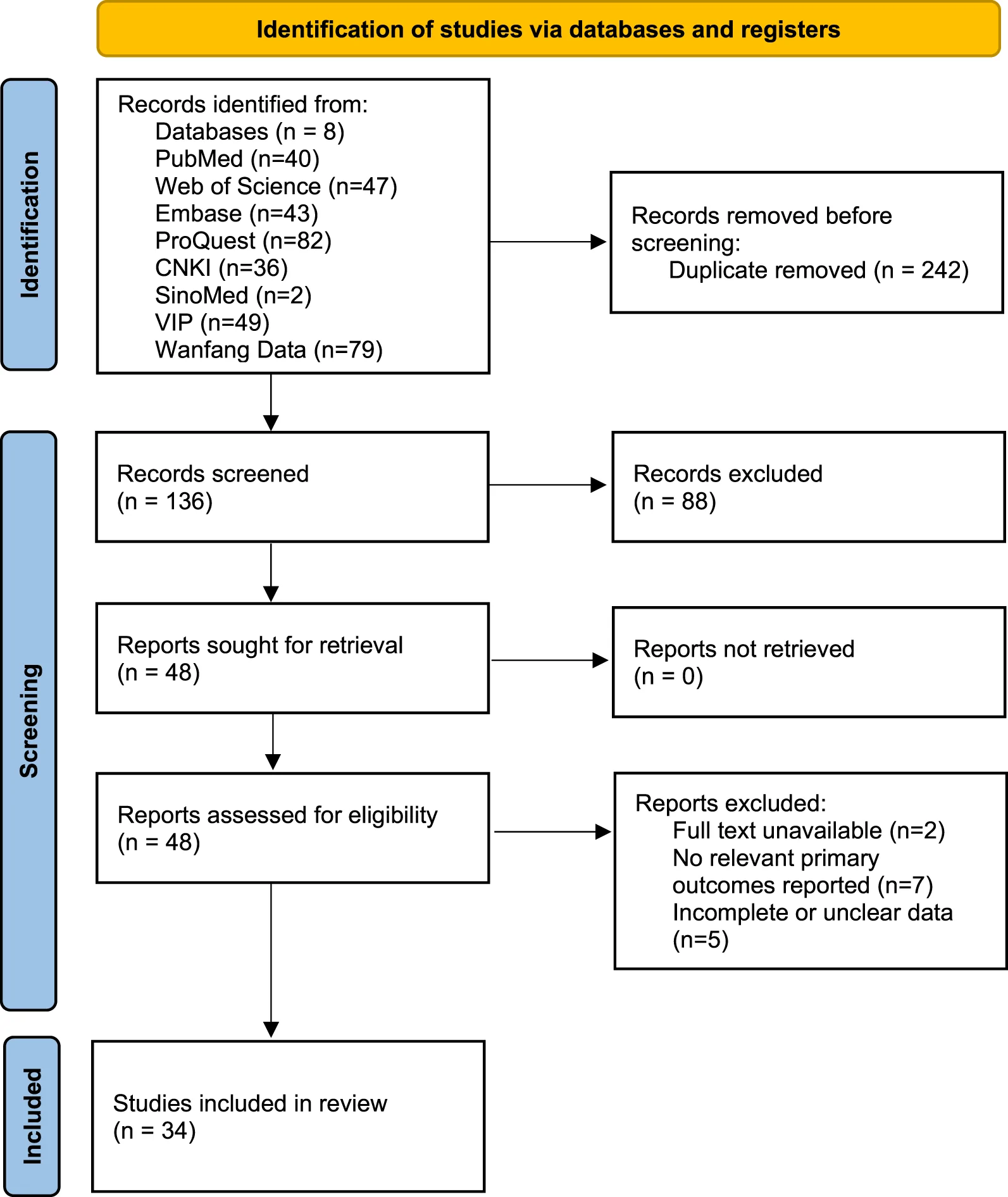
Flow chart of selection for studies in inclusion.

A total of 378 potentially relevant records were identified across eight databases: PubMed (n = 40), WoS (n = 47), Embase (n = 43), ProQuest (n = 82), CNKI (n = 36), SinoMed (n = 2), VIP (n = 49), and Wanfang Data (n = 79). After removing 242 duplicate records, 136 articles remained for initial screening. Subsequent screening of titles and abstracts led to the exclusion of 88 records based on the following criteria: reviews, comments, or editorials (n = 41); combined interventions with other drugs or decoctions (n = 19); *in vitro* studies (n = 15); non-renal fibrosis models (n = 11); and clinical studies (n = 2). The remaining 48 articles underwent full-text review, which resulted in the further exclusion of 14 studies due to unavailable full texts (n = 2), lack of relevant primary outcomes (n = 7), or incomplete/unclear data (n = 5). Ultimately, 34 eligible reports were included in the final systematic review (Yu et al., 2004; Zhu et al., 2014; Wang et al., 2014; Xu W. J. et al., 2014; Xu W. et al., 2014; Chen and Chen, 2016; Guo et al., 2016; Wang et al., 2017; Yang et al., 2017; Fan et al., 2017; Zhou et al., 2017; Yang et al., 2018; Song et al., 2018; Wang et al., 2018a; Wang et al., 2018b; Mao et al., 2019; Wang and Su, 2019; Wang et al., 2019; Zhang Y. et al., 2020; Zhou and Li, 2021; Gao et al., 2021; Jiang et al., 2022; Yuan et al., 2022; Zhang et al., 2022; Hu et al., 2022; Yu et al., 2022; Tang et al., 2022; Li D. et al., 2023; Li G. L. et al., 2023; Arda et al., 2024; Qing et al., 2024; Xu et al., 2025; Su and Song, 2025; He et al., 2025). The publication years of these included reports spanned from 2004 to 2025, indicating growing and extensive research interest in the renoprotective effects of AS-IV against renal fibrosis in recent years. To ensure data independence and prevent duplication bias across Chinese and English databases, we systematically screened reports sharing author groups or institutional affiliations for potential data overlap by cross-checking animal disease models, group sizes, administration routes, and experimental periods. Through this rigorous process, the publications by Wang et al., 2018a, Wang et al., 2018b; Wang et al., 2019 were identified as multiple reports derived from the same *in vivo* experimental cohort, as they shared identical baseline characteristics and quantitative outcome data (e.g., proteinuria and UACR). To avoid redundant statistical weighting, only the primary dataset (Wang et al., 2019) was included in the meta-analysis for these shared outcomes. However, because these three publications investigated completely distinct mechanistic pathways, all were retained in the qualitative synthesis to thoroughly reflect the pleiotropic mechanisms of AS-IV in attenuating renal fibrosis. The detailed literature selection process is illustrated in Figure 1.

### 3.2 Characteristics of included studies

A total of 34 studies were included in this review, comprising 18 published in Chinese (Yu et al., 2004; Zhu et al., 2014; Xu W. J. et al., 2014; Chen and Chen, 2016; Wang et al., 2017; Yang et al., 2017; Fan et al., 2017; Yang et al., 2018; Wang and Su, 2019; Zhou and Li, 2021; Jiang et al., 2022; Yuan et al., 2022; Zhang et al., 2022; Li G. L. et al., 2023; Qing et al., 2024; Xu et al., 2025; Su and Song, 2025; He et al., 2025), and 16 in English (Wang et al., 2014; Xu W. et al., 2014; Guo et al., 2016; Zhou et al., 2017; Song et al., 2018; Wang et al., 2018a; Wang et al., 2018b; Mao et al., 2019; Wang et al., 2019; Zhang Y. et al., 2020; Gao et al., 2021; Hu et al., 2022; Yu et al., 2022; Tang et al., 2022; Li D. et al., 2023; Arda et al., 2024). The publication years ranged from 2004 to 2025, with 18 studies published prior to 2020 (Yu et al., 2004; Zhu et al., 2014; Wang et al., 2014; Xu W. J. et al., 2014; Xu W. et al., 2014; Chen and Chen, 2016; Guo et al., 2016; Wang et al., 2017; Yang et al., 2017; Fan et al., 2017; Zhou et al., 2017; Yang et al., 2018; Song et al., 2018; Wang et al., 2018a; Wang et al., 2018b; Mao et al., 2019; Wang and Su, 2019; Wang et al., 2019), and the remaining 16 published between 2020 and 2025 (Zhang Y. et al., 2020; Zhou and Li, 2021; Gao et al., 2021; Jiang et al., 2022; Yuan et al., 2022; Zhang et al., 2022; Hu et al., 2022; Yu et al., 2022; Tang et al., 2022; Li D. et al., 2023; Li G. L. et al., 2023; Arda et al., 2024; Qing et al., 2024; Xu et al., 2025; Su and Song, 2025; He et al., 2025). A total of 769 animals were used across the included studies, divided into treatment (n = 466) and model (n = 303) groups. All subjects were either rats or mice. Specifically, 13 studies utilized Sprague-Dawley rats (n = 307) (Yu et al., 2004; Zhu et al., 2014; Wang et al., 2014; Chen and Chen, 2016; Mao et al., 2019; Wang and Su, 2019; Zhang Y. et al., 2020; Jiang et al., 2022; Yuan et al., 2022; Yu et al., 2022; Li D. et al., 2023; Li G. L. et al., 2023; He et al., 2025), 11 used C57BL/6 mice (n = 253) (Xu W. J. et al., 2014; Xu W. et al., 2014; Yang et al., 2017; Zhou et al., 2017; Yang et al., 2018; Song et al., 2018; Zhou and Li, 2021; Gao et al., 2021; Tang et al., 2022; Xu et al., 2025; Su and Song, 2025), 2 used KKAy mice (n = 44) (Wang et al., 2017; Wang et al., 2019), 3 used Wistar rats (n = 69) (Fan et al., 2017; Zhang et al., 2022; Arda et al., 2024), 2 used db/db mice (n = 80) (Guo et al., 2016; Hu et al., 2022), and 1 used MRL/lpr mice (n = 16) (Qing et al., 2024). Regarding the induction of renal fibrosis, 13 studies employed DKD models (Chen and Chen, 2016; Guo et al., 2016; Wang et al., 2017; Fan et al., 2017; Song et al., 2018; Mao et al., 2019; Wang and Su, 2019; Wang et al., 2019; Zhang Y. et al., 2020; Yuan et al., 2022; Hu et al., 2022; Li G. L. et al., 2023; Su and Song, 2025), eight utilized UUO models (Zhu et al., 2014; Wang et al., 2014; Xu W. J. et al., 2014; Xu W. et al., 2014; Yang et al., 2017; Zhou et al., 2017; Zhou and Li, 2021; He et al., 2025), and 4 used ischemia-reperfusion injury (IRI) models (Yu et al., 2004; Yang et al., 2018; Tang et al., 2022; Xu et al., 2025). The remaining seven studies involved other forms of renal fibrosis (Gao et al., 2021; Jiang et al., 2022; Zhang et al., 2022; Yu et al., 2022; Li D. et al., 2023; Arda et al., 2024; Qing et al., 2024), such as those induced by nephrotoxic drugs (e.g., cisplatin and tacrolimus). Among the included studies, 22 reported the purity of the AS-IV used (80%–98%), while the remaining 12 did not disclose this information. For intervention protocols, AS-IV was administered via intragastric gavage in 29 studies (Yu et al., 2004; Zhu et al., 2014; Wang et al., 2014; Xu W. J. et al., 2014; Chen and Chen, 2016; Guo et al., 2016; Wang et al., 2017; Yang et al., 2017; Fan et al., 2017; Zhou et al., 2017; Yang et al., 2018; Mao et al., 2019; Wang and Su, 2019; Wang et al., 2019; Zhang Y. et al., 2020; Zhou and Li, 2021; Gao et al., 2021; Jiang et al., 2022; Yuan et al., 2022; Zhang et al., 2022; Hu et al., 2022; Yu et al., 2022; Tang et al., 2022; Li D. et al., 2023; Li G. L. et al., 2023; Qing et al., 2024; Xu et al., 2025; Su and Song, 2025; He et al., 2025), via intraperitoneal injection in 2 studies (Xu W. et al., 2014; Arda et al., 2024), and as a dietary supplement in 1 study (Song et al., 2018). Among the 15 studies involving intragastric administration in rats (Yu et al., 2004; Zhu et al., 2014; Wang et al., 2014; Chen and Chen, 2016; Fan et al., 2017; Mao et al., 2019; Wang and Su, 2019; Zhang Y. et al., 2020; Jiang et al., 2022; Yuan et al., 2022; Yu et al., 2022; Zhang et al., 2022; Li D. et al., 2023; Li G. L. et al., 2023; He et al., 2025), the tested or maximum administered doses ranged from 1.5 to 80 mg/kg/d (most frequently 40 mg/kg/d), with treatment durations spanning from 6 days to 12 weeks (most frequently 2 and 8 weeks). In the 14 studies involving intragastric administration in mice (Xu W. J. et al., 2014; Guo et al., 2016; Wang et al., 2017; Yang et al., 2017; Zhou et al., 2017; Yang et al., 2018; Wang et al., 2019; Zhou and Li, 2021; Gao et al., 2021; Hu et al., 2022; Tang et al., 2022; Qing et al., 2024; Xu et al., 2025; Su and Song, 2025), doses ranged from 10 to 120 mg/kg/d (most frequently 20 and 40 mg/kg/d), administered over 1–12 weeks (most frequently 2 weeks). Histopathological evaluations were conducted in 31 studies, including hematoxylin and eosin staining (n = 25), Masson’s trichrome staining (n = 23), Periodic Acid-Schiff staining (n = 10), and Sirius Red staining (n = 3). For primary outcomes, Scr, BUN, and proteinuria were reported in 26, 22, and 11 studies, respectively. Additionally, TGF-β1, α-SMA, and fibronectin expression were documented in 14, 12, and 16 studies, respectively. Regarding secondary outcomes, seven studies reported collagen area or scores based on Masson’s staining, while another five reported the UACR. In addition, various studies reported markers related to fibrosis (type I and IV collagen, E-cadherin, p-Smad3, and Smad7), inflammation (TNF-α, IL-1β, IL-6, and NF-κB), and oxidative stress (MDA and HO-1). The characteristics of the included studies are presented in Supplementary Table S2. Detailed information on AS-IV used in each study is shown in Supplementary Table S3.

### 3.3 Study quality

**FIGURE 2:**
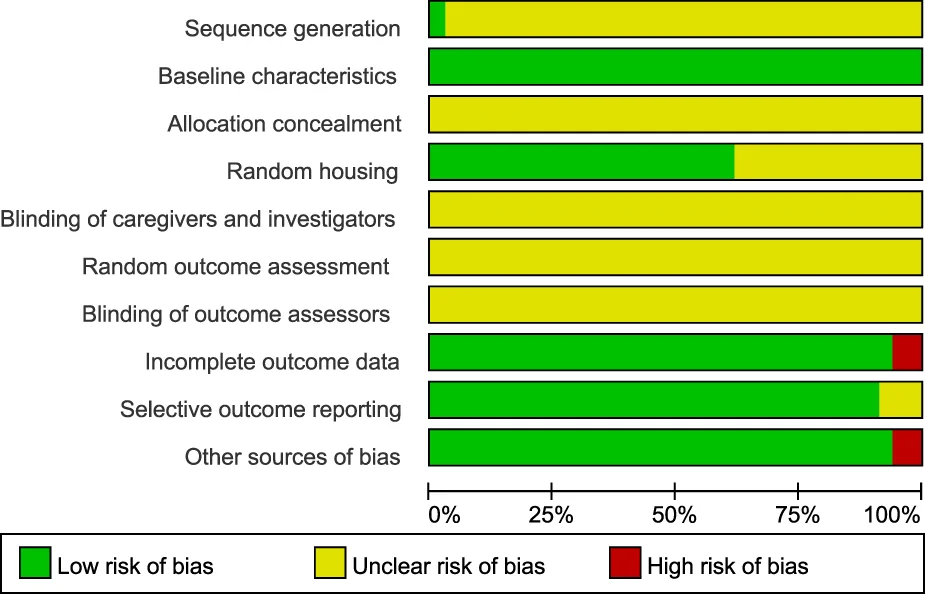
Risk of bias and quality assessment of the included studies.

**FIGURE 3:**
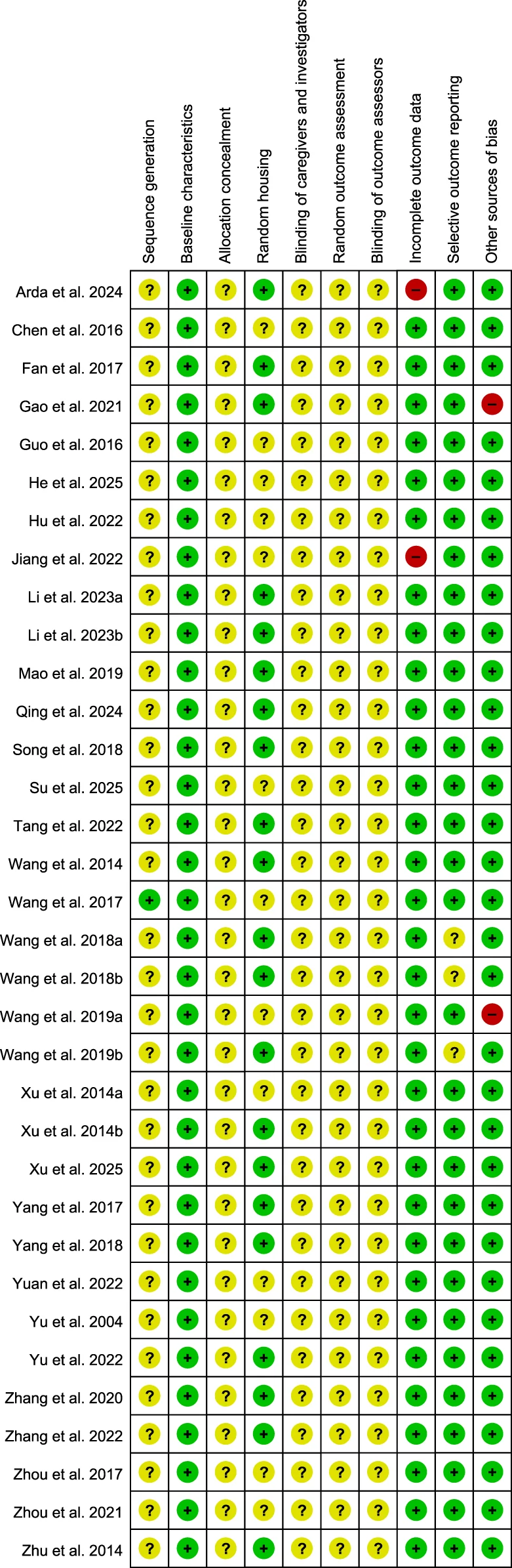
Summary of risk of bias and quality assessment of the included studies.

The methodological quality of the included studies was evaluated using the SYRCLE risk of bias tool. Regarding sequence generation, only one of the 34 included studies reported a specific randomization method (Wang et al., 2017). Thirty studies mentioned random allocation without detailing the specific procedure, while the remaining three failed to specify whether the animals were randomized. Twenty-one studies explicitly described identical housing conditions, three solely mentioned maintaining the animals in a specific pathogen-free environment, and ten completely omitted housing details. Although all studies demonstrated balanced baseline characteristics across groups, none reported allocation concealment or random housing. Furthermore, the implementation of blinding for caregivers or investigators was not reported in any of the studies. Incomplete outcome data were identified in two studies due to animal mortality during the experiments (Jiang et al., 2022; Arda et al., 2024), with no explanation provided regarding the potential impact of these missing data on the final outcomes. Regarding the specific domain of selective outcome reporting, three studies presented electron microscopy findings in the Results section without outlining corresponding experimental protocols in the Methods section, resulting in an unclear risk of bias for this domain (Wang et al., 2018a; Wang et al., 2018b; Wang et al., 2019). Other sources of bias were observed in two studies: one utilized incorrect data units (Wang and Su, 2019), and another required a post-publication correction (Gao et al., 2021). The overall and study-specific methodological quality assessments are illustrated in Figures 2, 3, respectively.

### 3.4 Effectiveness

#### 3.4.1 Primary outcomes

##### 3.4.1.1 Scr

**FIGURE 4:**
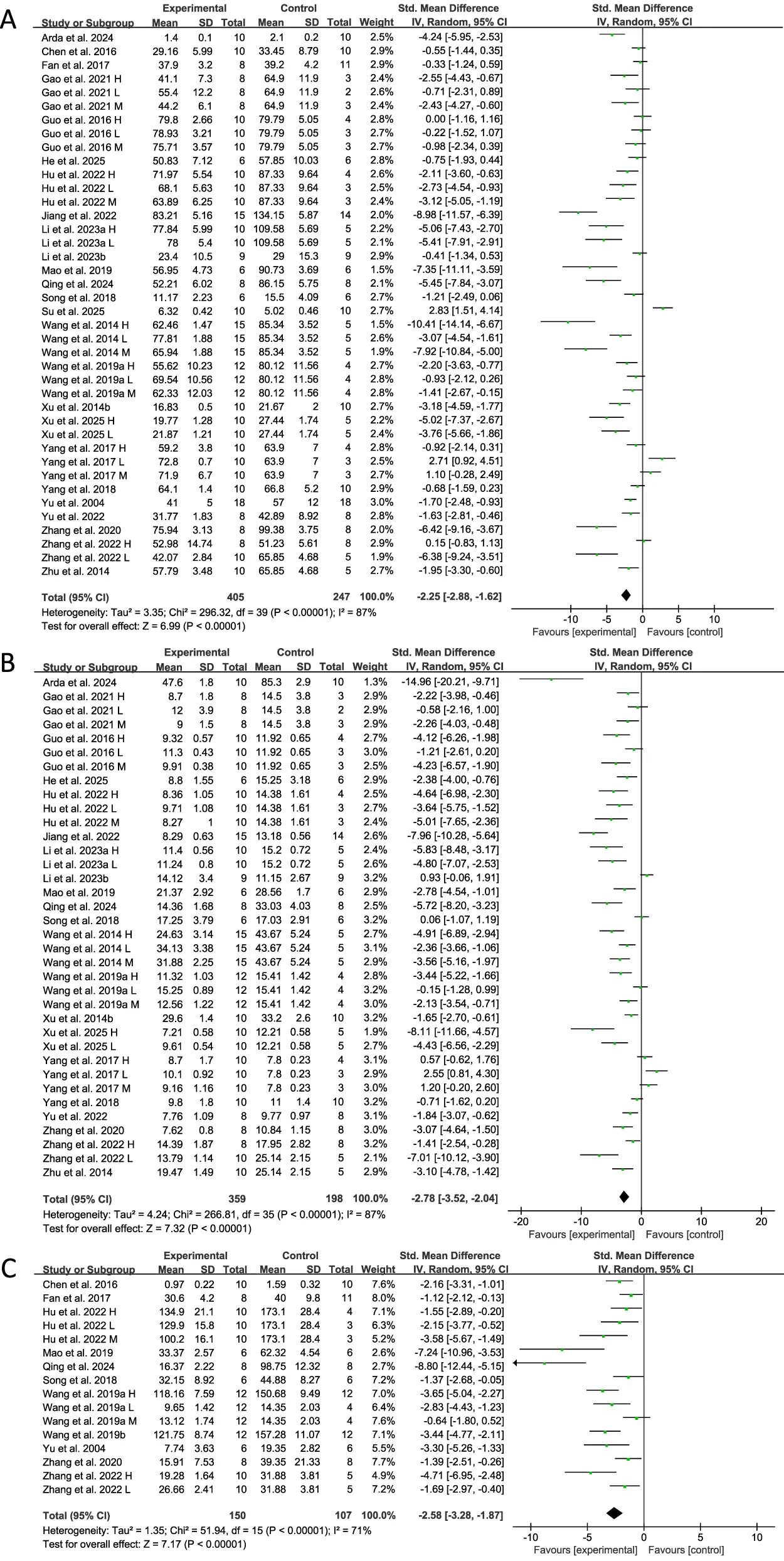
Forest plot: effect of AS-IV on **(A)** Scr, **(B)** BUN, **(C)** proteinuria.

Pooled analysis of 26 studies (Yu et al., 2004; Wang et al., 2014; Zhu et al., 2014; Xu W. et al., 2014; Chen and Chen, 2016; Guo et al., 2016; Fan et al., 2017; Yang et al., 2017; Song et al., 2018; Yang et al., 2018; Mao et al., 2019; Wang and Su, 2019; Zhang Y. et al., 2020; Gao et al., 2021; Hu et al., 2022; Jiang et al., 2022; Yuan et al., 2022; Yu et al., 2022; Zhang et al., 2022; Li D. et al., 2023; Li G. L. et al., 2023; Arda et al., 2024; Qing et al., 2024; He et al., 2025; Su and Song, 2025; Xu et al., 2025) showed that AS-IV significantly reduced Scr levels in renal fibrosis animal models relative to control groups (n = 652; SMD, −2.25; 95% CI, −2.88 to −1.62; heterogeneity: χ2 = 296.32, *P* < 0.00001, *I* ^*2*^ = 87%, Figure 4A).

##### 3.4.1.2 BUN

Similarly, data synthesized from 22 studies (Zhu et al., 2014; Wang et al., 2014; Xu W. et al., 2014; Guo et al., 2016; Yang et al., 2017; Song et al., 2018; Yang et al., 2018; Mao et al., 2019; Wang and Su, 2019; Zhang Y. et al., 2020; Gao et al., 2021; Yu et al., 2022; Zhang et al., 2022; Jiang et al., 2022; Yuan et al., 2022; Hu et al., 2022; Li D. et al., 2023; Li G. L. et al., 2023; Arda et al., 2024; Qing et al., 2024; Xu et al., 2025; He et al., 2025) demonstrated a significant decrease in BUN levels following AS-IV intervention (n = 557; SMD, −2.78; 95% CI, −3.52 to −2.04; heterogeneity: χ2 = 266.81, *P* < 0.00001, *I* ^*2*^ = 87%, Figure 4B).

##### 3.4.1.3 Proteinuria

Regarding proteinuria, a meta-analysis of 11 studies (Yu et al., 2004; Chen and Chen, 2016; Fan et al., 2017; Song et al., 2018; Mao et al., 2019; Wang and Su, 2019; Wang et al., 2019; Zhang Y. et al., 2020; Hu et al., 2022; Zhang et al., 2022; Qing et al., 2024) indicated that AS-IV exerted a pronounced antiproteinuric effect compared to control groups (n = 257; SMD, −2.58; 95% CI, −3.28 to −1.87; heterogeneity: χ2 = 51.94, *P* < 0.00001, *I* ^*2*^ = 71%, Figure 4C).

##### 3.4.1.4 TGF-β1

**FIGURE 5:**
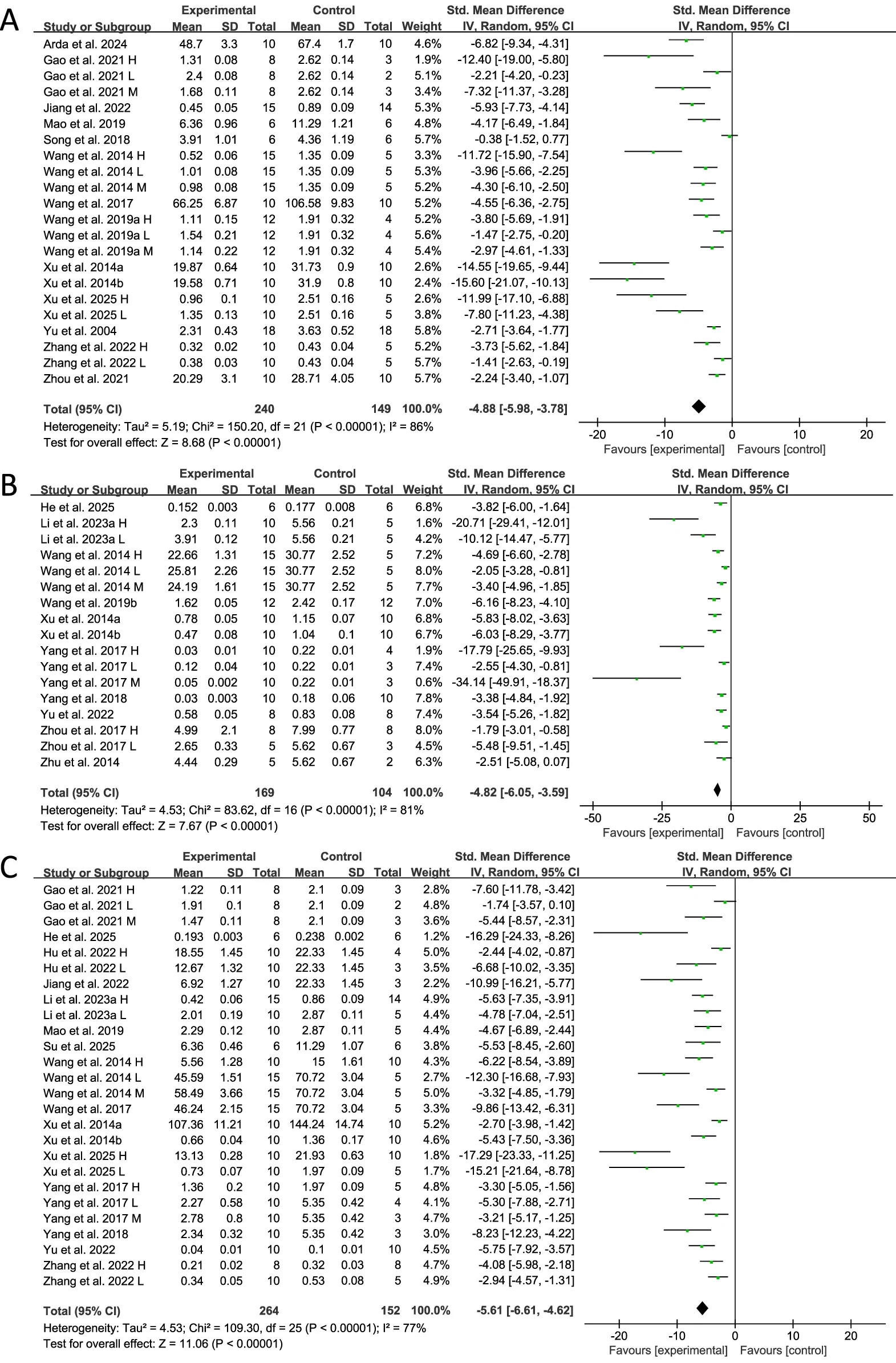
Forest plot: effect of AS-IV on **(A)** TGF-β1, **(B)** fibronectin, **(C)** α-SMA.

Expression levels of TGF-β1 were evaluated in 14 studies (Yu et al., 2004; Wang et al., 2014; Xu W. J. et al., 2014; Xu W. et al., 2014; Wang et al., 2017; Song et al., 2018; Mao et al., 2019; Wang and Su, 2019; Gao et al., 2021; Zhou and Li, 2021; Jiang et al., 2022; Zhang et al., 2022; Arda et al., 2024; Xu et al., 2025). The pooled results confirmed that AS-IV markedly suppressed TGF-β1 expression (n = 389; SMD, −4.88; 95% CI, −5.98 to −3.78; heterogeneity: χ2 = 150.2, *P* < 0.00001, *I* ^*2*^ = 86%, Figure 5A).

##### 3.4.1.5 Fibronectin

Pooled analysis of 12 studies (Wang et al., 2014; Zhu et al., 2014; Xu W. J. et al., 2014; Xu W. et al., 2014; Yang et al., 2017; Zhou et al., 2017; Yang et al., 2018; Wang et al., 2019; Zhang Y. et al., 2020; Yu et al., 2022; Li D. et al., 2023; He et al., 2025) revealed that AS-IV treatment significantly downregulated fibronectin levels in animal models of renal fibrosis compared to control groups (n = 273; SMD, −4.82; 95% CI, −6.05 to −3.59; heterogeneity: χ2 = 83.62, *P* < 0.00001, *I* ^*2*^ = 81%, Figure 5B).

##### 3.4.1.6 α-SMA

Similarly, data from 16 studies (Wang et al., 2014; Xu W. J. et al., 2014; Xu W. et al., 2014; Wang et al., 2017; Yang et al., 2017; Yang et al., 2018; Mao et al., 2019; Gao et al., 2021; Hu et al., 2022; Jiang et al., 2022; Yu et al., 2022; Zhang et al., 2022; Li D. et al., 2023; He et al., 2025; Su and Song, 2025; Xu et al., 2025) verified that AS-IV significantly downregulated α-SMA levels (n = 416; SMD, −5.61; 95% CI, −6.61 to −4.62; heterogeneity: χ2 = 109.3, *P* < 0.00001, *I* ^*2*^ = 77%, Figure 5C).

#### 3.4.2 Secondary outcomes

##### 3.4.2.1 Renal histopathology

**FIGURE 6:**
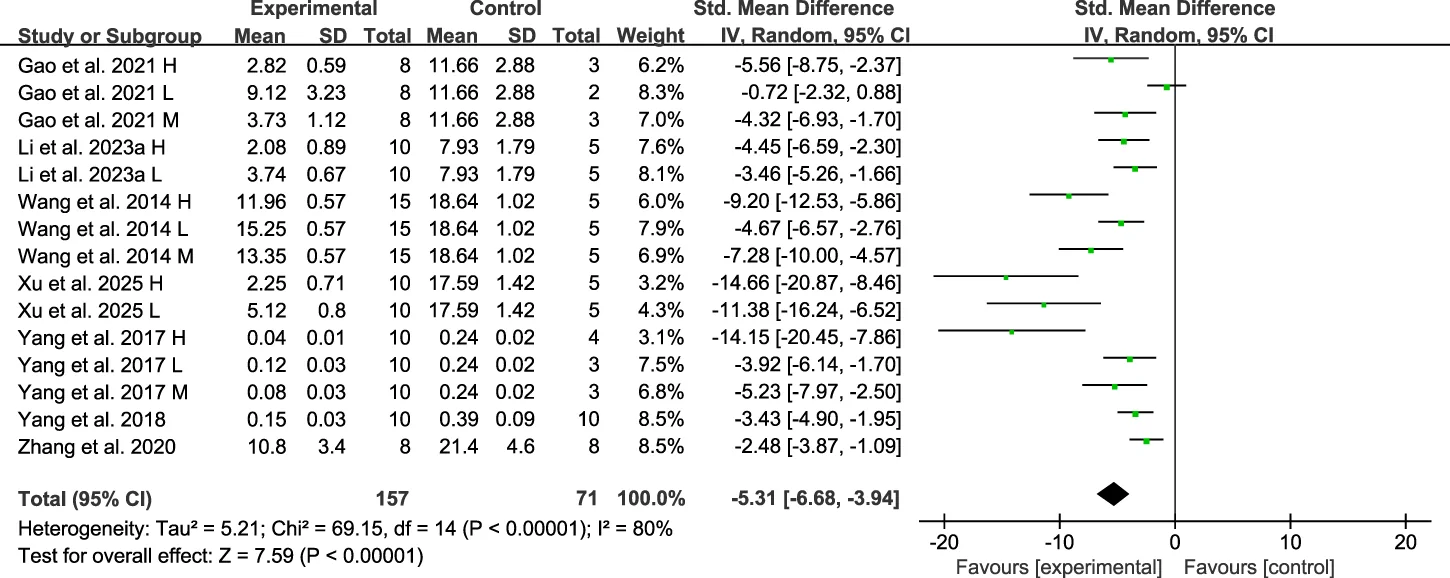
Forest plot: effect of AS-IV on renal histopathology.

Based on data from seven studies (Wang et al., 2014; Yang et al., 2017; Yang et al., 2018; Zhang Y. et al., 2020; Gao et al., 2021; Li D. et al., 2023; Xu et al., 2025), AS-IV treatment substantially attenuated the collagen area or histological scores determined by Masson’s trichrome staining (n = 228; SMD, −5.31; 95% CI, −6.68 to −3.94; heterogeneity: χ2 = 69.15, *P* < 0.00001, *I* ^*2*^ = 80%, Figure 6).

##### 3.4.2.2 UACR

**FIGURE 7:**
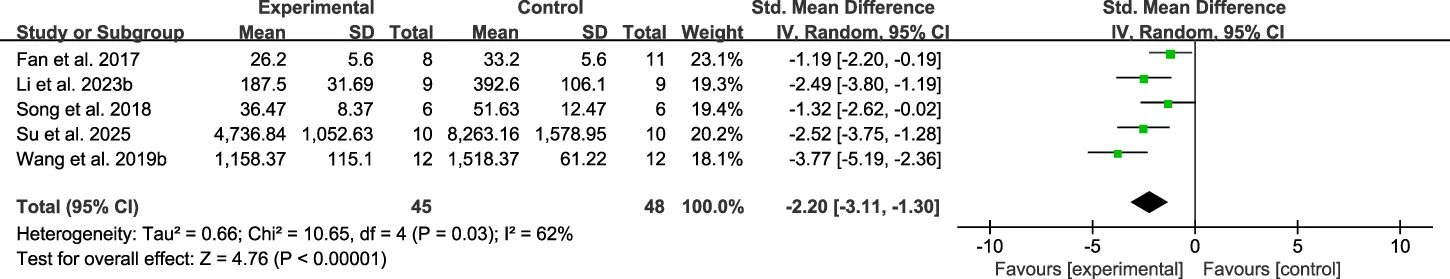
Forest plot: effect of AS-IV on UACR.

A meta-analysis of five studies (Fan et al., 2017; Song et al., 2018; Wang et al., 2019; Li G. L. et al., 2023; Su and Song, 2025) indicated a significant reduction in the UACR among AS-IV-treated animals relative to control groups (n = 93; SMD, −2.20; 95% CI, −3.11 to −1.3; heterogeneity: χ2 = 10.65, *P* < 0.00001, *I* ^*2*^ = 62%, Figure 7).

##### 3.4.2.3 Biomarkers of renal fibrosis

**FIGURE 8:**
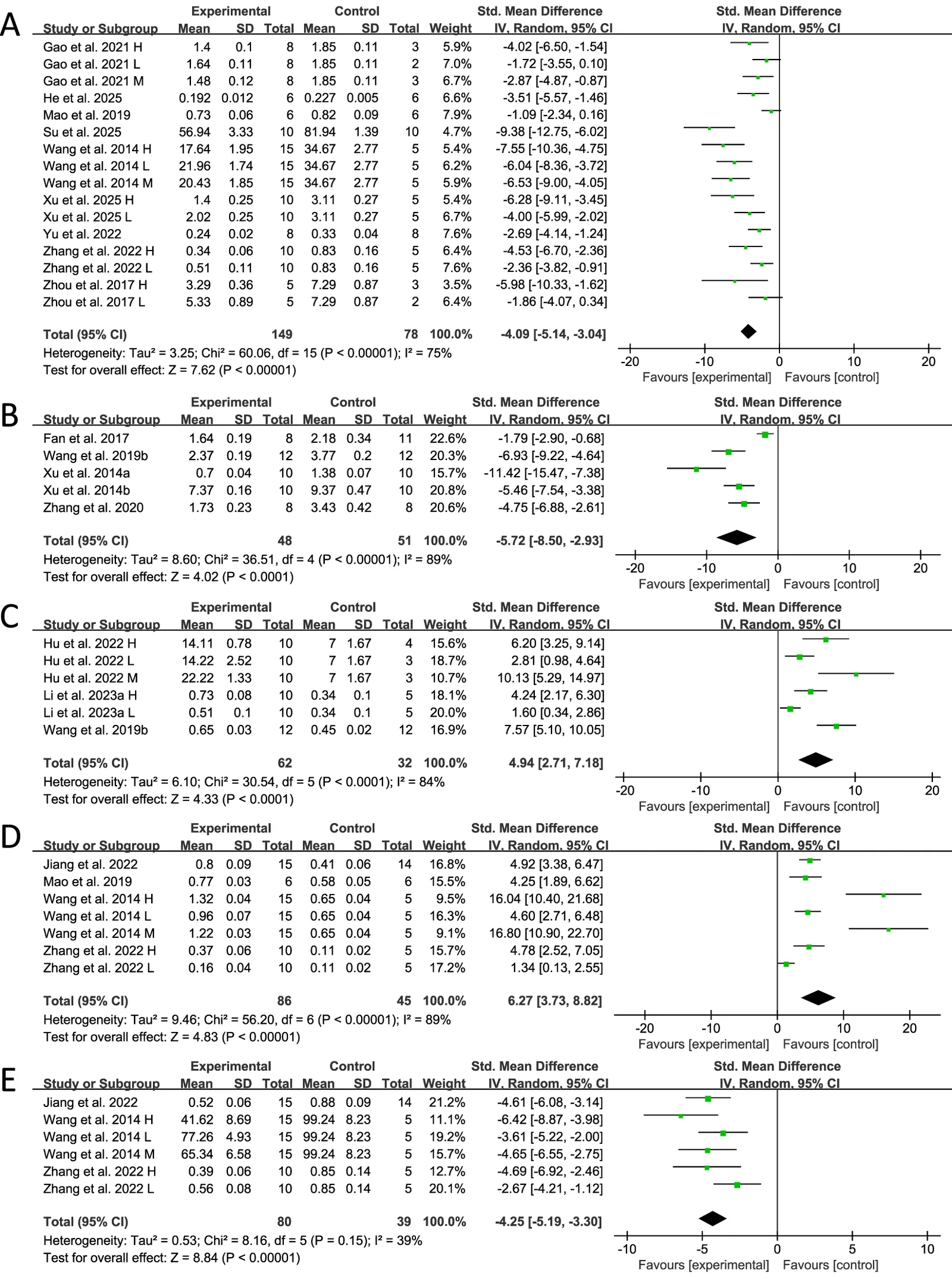
Forest plot: effect of AS-IV on **(A)** type I collagen, **(B)** type IV collagen, **(C)** E-cadherin, **(D)** Smad 7, **(E)** p-Smad3.

The effect of AS-IV in modulating specific fibrotic markers was further corroborated. Pooled analyses demonstrated significant reductions in type I collagen across nine studies (Wang et al., 2014; Zhou et al., 2017; Mao et al., 2019; Gao et al., 2021; Yu et al., 2022; Zhang et al., 2022; He et al., 2025; Su and Song, 2025; Xu et al., 2025) (n = 227; SMD, −4.09; 95% CI, −5.14 to −3.04; heterogeneity: χ2 = 60.06, *P* < 0.00001, *I* ^*2*^ = 75%, Figure 8A), and type IV collagen across five studies (Xu W. J. et al., 2014; Xu W. et al., 2014; Fan et al., 2017; Wang et al., 2019; Zhang Y. et al., 2020) (n = 99; SMD, −5.72; 95% CI, −8.5 to −2.93; heterogeneity: χ2 = 36.51, *P* < 0.0001, *I* ^*2*^ = 89%, Figure 8B). Furthermore, AS-IV significantly increased the expression of E-cadherin (Wang et al., 2019; Hu et al., 2022; Li D. et al., 2023) (n = 94; SMD, 4.94; 95% CI, 2.71 to 7.18; heterogeneity: χ2 = 30.54, *P* < 0.0001, *I* ^*2*^ = 84%, Figure 8C) and Smad 7 (Wang et al., 2014; Mao et al., 2019; Jiang et al., 2022; Zhang et al., 2022) (n = 131; SMD, 6.27; 95% CI, 3.73 to 8.82; heterogeneity: χ2 = 56.2, *P* < 0.00001, *I* ^*2*^ = 89%, Figure 8D), while notably decreasing p-Smad3 levels (Wang et al., 2014; Jiang et al., 2022; Zhang et al., 2022) (n = 119; SMD, −4.25; 95% CI, −5.19 to −3.3; heterogeneity: χ2 = 8.16, *P* < 0.00001, *I* ^*2*^ = 39%, Figure 8E).

##### 3.4.2.4 Oxidative stress index

**FIGURE 9:**
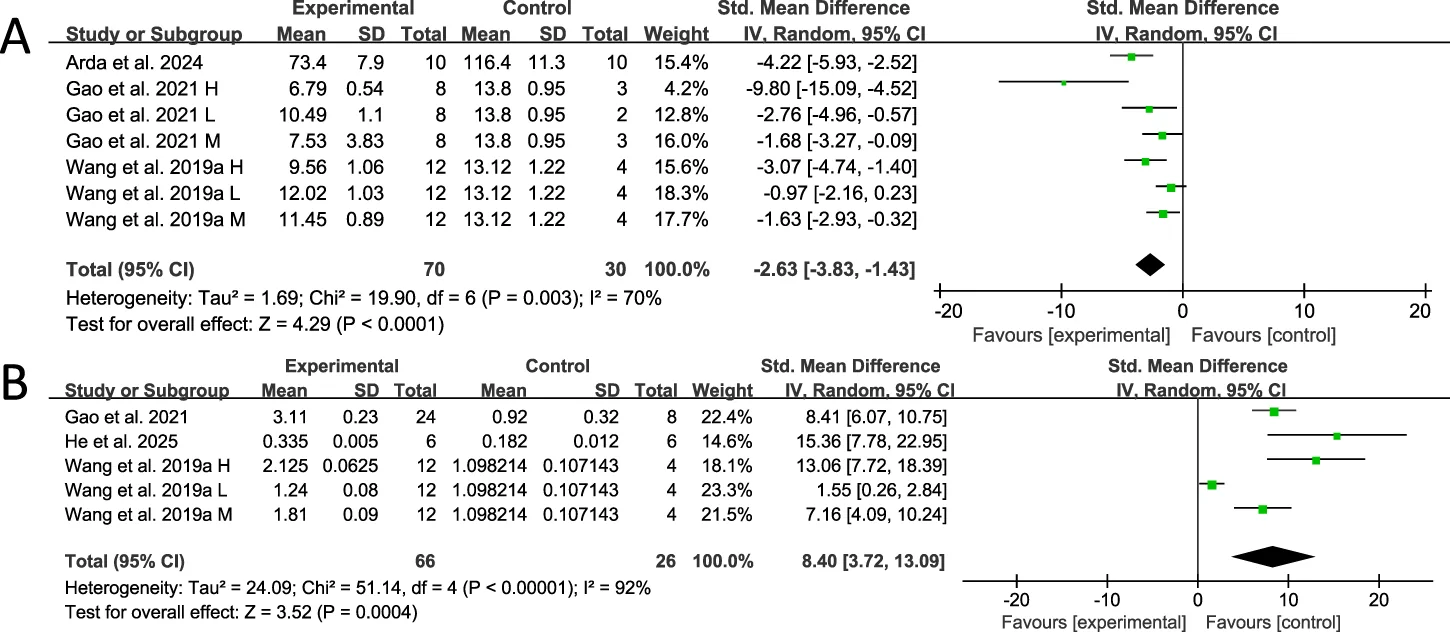
Forest plot: effect of AS-IV on **(A)** MDA, **(B)** HO-1.

Pooled analysis of 3 studies (Wang and Su, 2019; Gao et al., 2021; Arda et al., 2024) showed that AS-IV significantly reduced MDA levels in renal fibrosis animal models relative to control groups (n = 100; SMD, −2.63; 95% CI, −3.83 to −1.43; heterogeneity: χ2 = 19.9, *P* < 0.0001, *I* ^*2*^ = 70%, Figure 9A). Pooled analysis of 3 studies (Wang and Su, 2019; Gao et al., 2021; He et al., 2025) showed that AS-IV significantly increased HO-1 levels in renal fibrosis animal models relative to control groups (n = 92; SMD, 8.4; 95% CI, 3.72 to 13.09; heterogeneity: χ2 = 51.14, *P* = 0.0004, *I* ^*2*^ = 92%, Figure 9B).

##### 3.4.2.5 Inflammation levels

**FIGURE 10:**
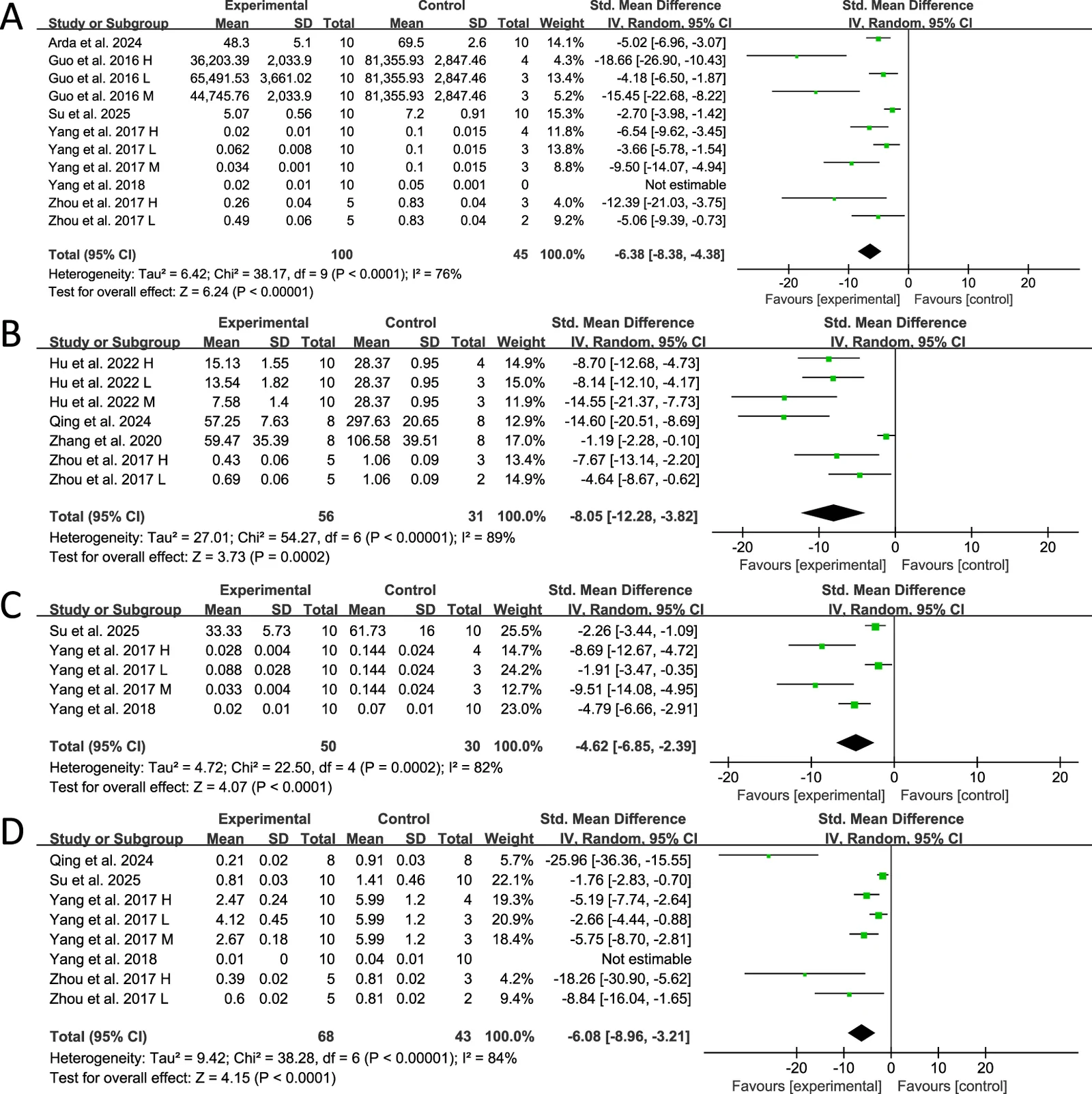
Forest plot: effect of AS-IV on **(A)** TNF-α, **(B)** IL-1β, **(C)** IL-6, **(D)** NF-κB.

AS-IV exhibited robust anti-inflammatory effects. Synthesized data revealed significant reductions in TNF-α (Yang et al., 2017; Zhou et al., 2017; Yang et al., 2018; Arda et al., 2024; Su and Song, 2025) (n = 145; SMD, −6.38; 95% CI, −8.38 to −4.38; heterogeneity: χ2 = 38.17, *P* < 0.00001, *I* ^*2*^ = 76%, Figure 10A), IL-1β (Zhou et al., 2017; Zhang Y. et al., 2020; Hu et al., 2022; Qing et al., 2024) (n = 87; SMD, −8.05; 95% CI, −12.28 to −3.82; heterogeneity: χ2 = 54.27, *P* = 0.0002, *I* ^*2*^ = 89%, Figure 10B), IL-6 (Yang et al., 2017; Yang et al., 2018; Su and Song, 2025) (n = 80; SMD, −4.62; 95% CI, −6.85 to −2.39; heterogeneity: χ2 = 22.5, *P* < 0.0001, *I* ^*2*^ = 82%, Figure 10C), and NF-κB levels compared to control groups (Yang et al., 2017; Zhou et al., 2017; Yang et al., 2018; Qing et al., 2024; Su and Song, 2025) (n = 111; SMD, −6.08; 95% CI, −8.96 to −3.21; heterogeneity: χ2 = 38.28, *P* < 0.0001, *I* ^*2*^ = 84%, Figure 10D).

### 3.5 Sensitivity analysis

**FIGURE 11:**
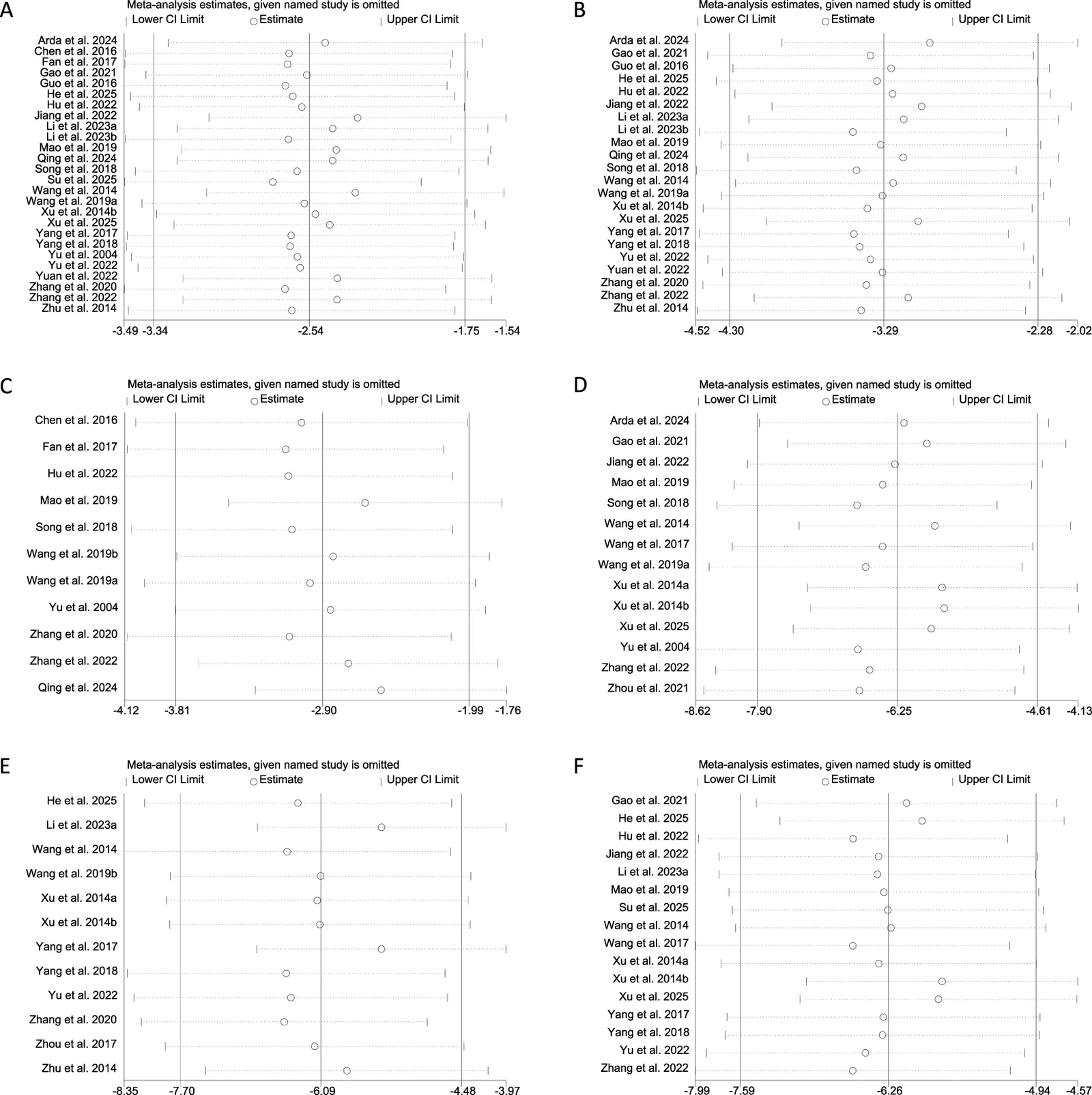
Sensitivity analysis charts on **(A)** Scr, **(B)** BUN, **(C)** Proteinuria, **(D)** TGF-β1, **(E)** Fibronectin, **(F)** α-SMA.

To evaluate the stability of the meta-analysis, a leave-one-out sensitivity analysis was conducted by sequentially omitting individual studies and recalculating the pooled effect estimates. The findings indicated that the overall results remained consistent, as no single study disproportionately altered the magnitude or direction of the pooled effects. The sensitivity analysis results for the primary outcomes are illustrated in Figure 11.

### 3.6 Subgroup analysis

Subgroup analyses of the primary outcomes were conducted based on four predefined covariates: publication year, underlying animal disease model, intragastric dose, and treatment duration. The results indicated that the intragastric dose was a potential source of heterogeneity for Scr, whereas publication year likely accounted for the heterogeneity observed in BUN. Additionally, treatment duration emerged as a possible source of heterogeneity for TGF-β1, while both the underlying animal disease model and treatment duration contributed to the heterogeneity of α-SMA. Conversely, none of the four covariates explained the heterogeneity present in the analyses of proteinuria and fibronectin. Detailed subgroup analysis results are summarized in Supplementary Table S4.

### 3.7 Publication bias

**FIGURE 12:**
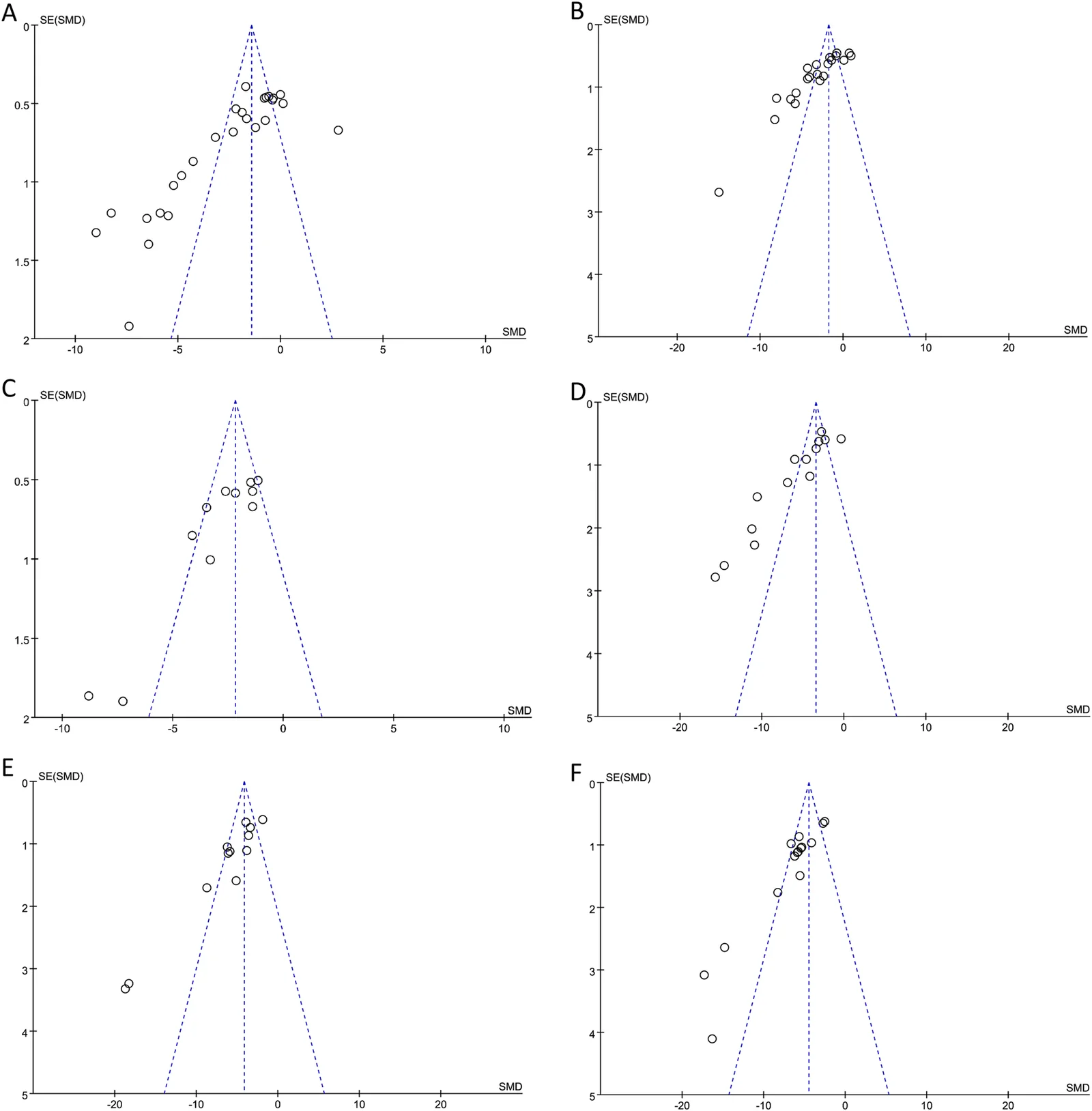
Funnel plots on **(A)** Scr, **(B)** BUN, **(C)** Proteinuria, **(D)** TGF-β1, **(E)** Fibronectin, **(F)** α-SMA.

Publication bias for the primary outcomes was assessed using funnel plots and Egger’s test. Visual inspection of the funnel plots revealed potential asymmetry, suggesting that publication bias could not be ruled out (Figure 12). This asymmetry may be attributed to factors such as the potential omission of unpublished negative findings or the suboptimal methodological quality of the included studies. These observations were further corroborated by the results of Egger’s test (Supplementary Table S5). To further evaluate the potential impact of this bias, a trim-and-fill analysis was performed. Except for TGF-β1, for which one missing study was estimated, no additional missing studies were identified for the other primary outcomes. In addition, the adjusted effect size for TGF-β1 remained nearly identical to the original estimate (Supplementary Table S6).

### 3.8 Dose-response analysis

The dose-response relationships of the primary outcomes were evaluated using SMDs to account for baseline variations across different animal models. Assessment via the restricted cubic spline model showed that Scr, BUN, and TGF-β1 exhibited linear dose-response associations. In contrast, proteinuria, fibronectin, and α-SMA showed significant non-linear dose-response relationships. Subsequent linear dose-response analyses indicated that Scr, BUN, and TGF-β1 possessed statistically significant negative linear associations with the treatment dose. For these parameters, the 95% CIs remained strictly below the zero reference line across the entire evaluated dose range, indicating that escalating doses of AS-IV led to a progressive and significant reduction in their levels. Detailed linear dose-response charts are presented in Supplementary Figure S1. Non-linear dose-response analyses of fibronectin and α-SMA similarly showed overall negative dose-dependent trends, suggesting that increasing AS-IV doses effectively diminished the expression levels of these markers. Regarding proteinuria, although categorical subgroup analyses showed that AS-IV significantly reduced proteinuria levels across all predefined dose tiers, the continuous non-linear dose-response curve exhibited an anomalous non-monotonic, inverted U-shaped trend. This deviation is likely attributable to the limited number of included studies for this specific parameter (n = 8) coupled with its inherently high inter-study heterogeneity. Detailed non-linear dose-response charts are presented in Supplementary Figure S2.

## 4 Discussion

### 4.1 Summary of the evidence

This study evaluated the protective effects of AS-IV in animal models of renal fibrosis. To the best of our knowledge, this is the first systematic review evaluating AS-IV across etiologically diverse models of renal fibrosis. We included 34 reports covering 19 outcomes. Because the included studies involved multiple etiological models of renal fibrosis, most outcomes exhibited considerable statistical heterogeneity. Methodologically, the overall quality of the included studies was suboptimal, primarily because the majority failed to strictly adhere to standard animal research reporting guidelines. This lack of compliance led to a large number of experimental details not being disclosed, which compromised the transparency and increased the risk of bias in these studies. Nevertheless, sensitivity analyses showed that no single study disproportionately influenced the pooled effect estimates of the primary outcomes. Subsequently, we explored potential sources of heterogeneity for the primary outcomes through four dimensions of subgroup analyses: intragastric dose was a source of heterogeneity for Scr; publication year for BUN; treatment duration for TGF-β1; and the underlying animal disease model alongside treatment duration for α-SMA. Regarding publication bias, visual inspection of funnel plots and Egger’s test indicated that such bias could not be ruled out for the six primary outcomes; however, subsequent trim-and-fill analyses revealed that no missing studies were imputed for five of these indicators. Still, we cannot definitively exclude the presence of publication bias, because the considerable experimental and methodological heterogeneity among the included studies may have compromised the performance of the trim-and-fill method. Dose-response analyses found that Scr, BUN, TGF-β1, fibronectin, and α-SMA showed overall negative dose-dependent trends. Conversely, the non-linear dose-response analysis of proteinuria displayed an unexpected non-monotonic, inverted U-shaped trend, with a peak slightly above 40 mg/kg. However, our subgroup analyses confirmed significant proteinuria reduction at each dose tier (≤30 mg/kg, > 30 and ≤ 60 mg/kg, and > 60 mg/kg). This discrepancy does not invalidate the subgroup findings; rather, the anomalous curve inflection likely reflects data sparsity and high heterogeneity. Therefore, despite these methodological and statistical limitations, our findings suggest that AS-IV holds renoprotective potential in animal models of renal fibrosis.

Among the primary outcomes, Scr and BUN are crucial clinical indices for evaluating renal function, and the accumulation of these nitrogenous wastes can promote the formation of a profibrotic microenvironment. Although previous studies indicate that nitrogenous wastes are suboptimal for reflecting the degree of fibrosis in early-stage kidney disease, they remain clinically relevant for assessing the severity of renal fibrosis in patients with CKD stages 3–5 (Wu et al., 2025; Chen et al., 2024). In the 26 and 22 studies reporting these respective outcomes, AS-IV significantly reduced Scr and BUN levels in animal models of renal fibrosis. Additionally, proteinuria is not only a universal biomarker of kidney disease but also a critical pathogenic factor driving renal injury; it actively participates in the pathophysiology of renal fibrosis, and its reduction helps alleviate fibrotic progression (Zoja et al., 2015). We found that AS-IV significantly ameliorated proteinuria across 11 included studies, and significantly lowered the UACR in five studies. As the most potent profibrotic cytokine and a key biomarker of organ fibrosis, TGF-β1 directly activates the expression of α-SMA and fibronectin via the TGF-β/Smad signaling pathway, thereby promoting the initiation and progression of renal fibrosis (Meng et al., 2016). Our findings show that AS-IV significantly decreased the expression of TGF-β1 and p-Smad3 while upregulating Smad7 and E-cadherin in animal models. As the classic marker for myofibroblasts, α-SMA serves as a core indicator of myofibroblast activation (Bauer et al., 2025). By evaluating the impact of AS-IV on α-SMA expression across 16 included studies, we found that AS-IV significantly inhibited its expression, indicating a pharmacological role in alleviating renal fibrosis by suppressing myofibroblast activation. Additionally, the preliminary assembly of fibronectin molecules is a biophysical prerequisite for the stable deposition of other ECM components (e.g., type I collagen) (Tomasini-Johansson et al., 2018). Consequently, its overexpression is acknowledged as a critical early event in the fibrotic pathological cascade. We found that AS-IV not only significantly inhibited fibronectin expression but also suppressed the expression of collagen components (type I and IV collagen). Therefore, synthesizing these findings with the corresponding histopathological assessments, we conclude that AS-IV exerts a robust inhibitory effect on renal fibrosis.

Through the analysis of the remaining secondary outcomes, we primarily explored the underlying mechanisms by which AS-IV alleviates renal fibrosis. Notably, several mechanistic markers—specifically E-cadherin, Smad7, p-Smad3, MDA, HO-1, IL-1β, and IL-6—were based on fewer than five studies each. Although pooling these small datasets is statistically feasible, the limited statistical power dictates that these specific results should be interpreted cautiously as exploratory evidence rather than definitive proof. Despite the modest number of included studies, these markers collectively outlined a consistent biological pattern that provides coherent mechanistic support for our anti-fibrotic outcomes. Consistent with previous preclinical research, our findings indicated that AS-IV significantly attenuated inflammation and oxidative stress, and ameliorated fibrotic indicators. A detailed discussion of these mechanisms is provided in the subsequent section.

### 4.2 Possible mechanism of action

CKD is a major global public health challenge, ranking as the ninth leading cause of death worldwide, driven largely by population aging and the rising prevalence of metabolic diseases (Francis et al., 2024). The initiation and progression of renal injury stem from diverse etiologies, including hypoxic, immune-mediated, and toxic injuries (Schreibing et al., 2023).

Although the past decade has witnessed considerable progress in elucidating the pathogenesis of CKD, the clinical translation of these novel insights requires further validation. Consequently, current clinical management primarily focuses on controlling the primary disease to mitigate renal injury and delay fibrotic progression (Djudjaj and Boor, 2019). For DKD—the leading cause of CKD—high-quality evidence-based medicine establishes that optimal glycemic control is the cornerstone for improving renal outcomes (Zoungas et al., 2017). Consistent with previous reports, our findings indicate that in DKD models, AS-IV not only improves renal function, evidenced predominantly by reductions in serum creatinine and blood urea nitrogen, but also exerts hypoglycemic effects (Supplementary Figure S3), thereby highlighting its therapeutic potential in the treatment of this specific etiology (Zhou et al., 2020).

Proteinuria is a universal biomarker of kidney disease and a critical pathological driver of renal injury and fibrosis (Zoja et al., 2015). Massive protein leakage into the urine induces protein toxicity, particularly precipitating tubulointerstitial injury. Under pathological conditions, filtered plasma proteins are excessively reabsorbed by proximal tubular epithelial cells via the megalin/cubilin receptor complex. This intracellular protein accumulation triggers pathophysiological processes such as lysosomal rupture, endoplasmic reticulum stress, and oxidative stress, leading to cellular damage and the subsequent release of profibrotic factors, ultimately culminating in tubulointerstitial fibrosis (Makhammajanov et al., 2024). Furthermore, regardless of the etiology or therapeutic modality, a reduction in proteinuria positively correlates with improved renal outcomes—a consensus strongly supported by extensive basic and clinical research (Longhitano et al., 2024). Our review reaffirms that AS-IV significantly reduces proteinuria in animal models of renal fibrosis, underscoring its broad-spectrum potential to mitigate renal injury and fibrogenesis.

During the initial stages of renal injury, transient matrix deposition and adaptive tissue repair facilitate wound healing. However, under conditions of chronic, sustained injury or maladaptive repair processes, the continuous synthesis of ECM outpaces its degradation (Schreibing et al., 2023). This imbalance progressively disrupts renal architecture—manifesting as tubular atrophy, glomerulosclerosis, and vascular rarefaction—thereby impairing normal renal function, driving disease progression, and ultimately leading to ESKD and associated mortality (Schreibing et al., 2023). Myofibroblasts, which exhibit phenotypic characteristics of both fibroblasts and smooth muscle cells, are the primary source of ECM during renal fibrogenesis (Meng et al., 2025). Under the stimulation of profibrotic factors, renal fibrosis is driven by a complex interplay of cellular responses: the strong activation of resident fibroblasts and pericytes into ECM-producing myofibroblasts, accompanied by the partial transdifferentiation of tubular epithelial and endothelial cells, which collectively orchestrate the local fibrotic microenvironment. Therefore, in addition to the aggressive management of primary diseases and proteinuria, mounting evidence supports targeting the local fibrotic microenvironment to inhibit myofibroblast activation and pathogenic cellular transdifferentiation as a broad strategy to halt renal fibrosis (Wang et al., 2019; Hu et al., 2022; Li et al., 2022; Li D. et al., 2023). The studies included in our review suggest that AS-IV possesses this therapeutic potential.

Oxidative stress and inflammatory responses are well-established pathological mechanisms driving CKD progression and act as critical links in creating a profibrotic microenvironment (Liu et al., 2025). Stimuli such as hyperglycemia, hyperlipidemia, hypertension, and nephrotoxins are frequently accompanied by varying degrees of oxidative stress and inflammation, thereby triggering renal injury (Wang and Zhang, 2024; Chae et al., 2023; Arendshorst et al., 2024; Motrenikova et al., 2025). In the kidney, these pathogenic factors increase reactive oxygen species (ROS) production by activating NADPH oxidases and inducing mitochondrial dysfunction (Lee and Jose, 2021). Simultaneously, they suppress antioxidant signaling pathways, downregulating antioxidant enzymes (e.g., superoxide dismutase [SOD]) and impairing ROS clearance, which results in toxic ROS accumulation (Lin et al., 2023). Beyond directly damaging cells via the oxidation of DNA, proteins, and lipids, ROS can activate pathways such as toll-like receptor (TLR)/myeloid differentiation factor 88 (MyD88), NF-κB, and TGF-β/Smad, further fueling inflammation and renal fibrosis (Qiu et al., 2023). According to the studies in our review (Wang and Su, 2019; Gao et al., 2021; Arda et al., 2024), AS-IV activates the nuclear factor E2-related factor 2 [NRF2]/HO-1 signaling pathway, inhibits ROS production, and upregulates the expression of antioxidant enzymes. Inflammation is a pivotal mechanism in the initiation and progression of fibrosis. A recent study confirmed that VCAM1^+^ICAM1^+^ inflammatory proximal tubule cells specifically localize to fibrotic niches. These cells play an active role in recruiting leukocytes and activating fibroblasts, correlating positively with the severity of fibrosis, which further reinforces the central role of inflammation in renal fibrogenesis (Reck et al., 2025). Functioning as a master transcriptional orchestrator of the inflammatory cascade, NF-κB undergoes nuclear translocation to initiate and amplify the downstream transcription of an extensive array of pro-inflammatory cytokines (e.g., TNF-α, IL-1β, IL-6), chemokines (e.g., monocyte chemoattractant protein-1), adhesion molecules (e.g., vascular cell adhesion molecule 1 [VCAM-1], intercellular adhesion molecule 1 [ICAM-1]), and TGF-β1 (Ren et al., 2024). This promotes leukocyte infiltration to the injury site via the classical rolling, firm adhesion, and transmigration cascade (Gerhardt and Ley, 2015). It is well known that inflammation characterized by monocyte/macrophage infiltration is a hallmark of renal injury (Zhang J. et al., 2020). Sensing the local microenvironment, macrophages predominantly polarize into two phenotypes: the pro-inflammatory M1 phenotype and the tissue-repairing M2 phenotype. During the early stages of physiological tissue injury, M1 macrophages dominate, exerting pro-inflammatory effects to clear damaged cells and pathogens. Following this phase, macrophages polarize toward the M2 phenotype to promote tissue repair and matrix remodeling, eventually resolving once repair is complete. However, under pathological conditions, persistent injury signals trigger chronic inflammation, leading to the sustained activation of M2 macrophages, aberrant tissue repair, and the progressive accumulation of ECM (Mack, 2018). Consequently, targeting macrophage polarization balance is a critical strategy for controlling renal fibrosis (Tang et al., 2019). Our review confirms that AS-IV suppresses the expression and nuclear translocation of NF-κB, decreases the expression and release of TNF-α, IL-1β, and IL-6, and mitigates fibrosis by inhibiting M1 polarization to attenuate initial inflammation during the early disease stages and by suppressing excessive M2 activation and infiltration during the later stages of injury (Tang et al., 2022; Su and Song, 2025).

Under physiological conditions, myofibroblasts facilitate tissue repair by producing ECM; however, once repair is complete, they typically undergo apoptosis to prevent excessive ECM deposition (Hinz and Lagares, 2020). In contrast, apoptosis in most terminally differentiated renal cells (e.g., endothelial cells and podocytes) promotes renal fibrosis, whereas autophagy confers anti-fibrotic protection (Lenoir et al., 2015). Specifically, in tubular epithelial cells, defective autophagy or impaired autophagic flux exacerbates G2/M cell cycle arrest, acting synergistically with apoptosis to accelerate fibrotic progression (Li et al., 2016). In glomerular mesangial cells, autophagy not only downregulates apoptosis but also directly sequesters and mediates the lysosomal degradation of abnormally accumulated type I collagen (Kim et al., 2012). Therefore, modulating the interplay between autophagy and apoptosis to concomitantly reduce collagen synthesis and enhance its degradation represents a promising strategy for controlling renal fibrosis. According to the studies in our review (Li D. et al., 2023; Wang et al., 2018a; Wang et al., 2018b), AS-IV shows this therapeutic potential.

**FIGURE 13:**
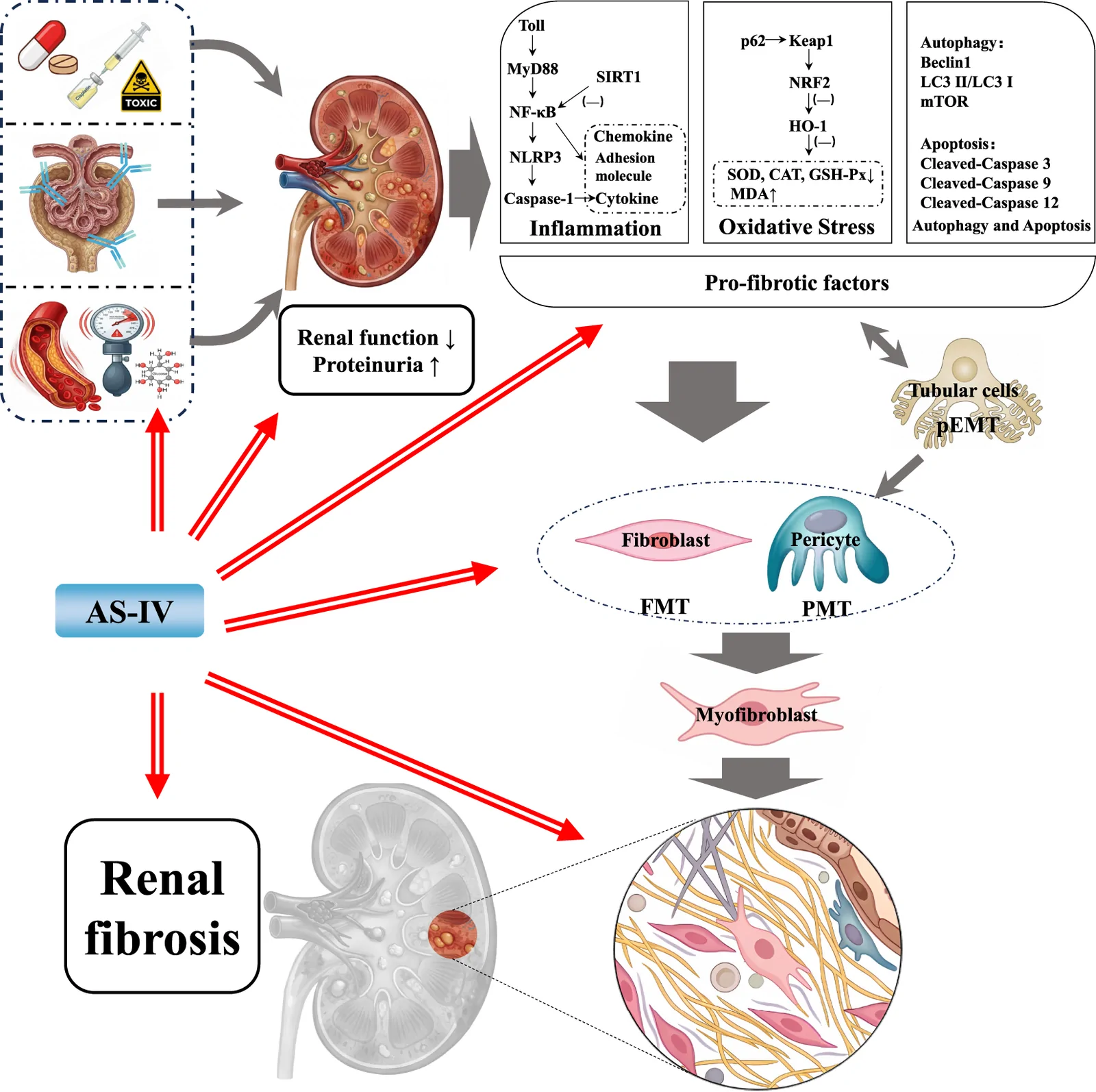
Potential mechanisms proposed for AS-IV action in animal models of renal fibrosis. Major etiologies of CKD, such as hypoxia, immune-mediated and toxic injuries, not only drive renal function decline and proteinuria but also induce inflammation, oxidative stress, and apoptosis, culminating in cellular damage. Persistent injury establishes a local profibrotic microenvironment within the kidney. Notably, injured tubular epithelial cells undergo pEMT and adopt a profibrotic secretory phenotype. Through strong paracrine signaling, these cells exacerbate the microenvironment, which subsequently drives the activation of resident fibroblasts and the transdifferentiation of pericytes into myofibroblasts. The resulting ECM deposition severely impairs normal renal architecture and function. AS-IV exerts multi-target renoprotective effects by mitigating primary pathological insults, reducing proteinuria, suppressing oxidative stress and inflammatory responses, and modulating autophagy and apoptosis. Through these integrated mechanisms, AS-IV effectively dismantles the profibrotic microenvironment and attenuates myofibroblast activation, ultimately ameliorating renal fibrosis. Acronyms: AS-IV—Astragaloside IV; CAT—catalase; CKD—chronic kidney disease; ECM—extracellular matrix; FMT—fibroblast to myofibroblast transition; GSH-Px—glutathione peroxidase; HO-1—heme oxygenase-1; Keap1—Kelch-like ECH-associated protein 1; LC3—microtubule-associated protein light chain 3; MDA—malondialdehyde; mTOR—mammalian target of rapamycin; MyD88—myeloid differentiation factor 88; NF-κB—nuclear factor κB; NLRP3—NOD-like receptor protein 3; NRF2—nuclear factor E2-related factor 2; pEMT—partial epithelial-mesenchymal transition; PMT—pericyte-myofibroblast transition; SIRT1—sirtuin 1; SOD—superoxide dismutase; TLR—Toll-like receptor.

Regarding specific signaling pathways, the TGF-β/Smad pathway remains the universally recognized master regulator of renal fibrosis. Although the specific inhibition of TGF-β1 has not achieved anticipated clinical efficacy—largely due to the pathway’s involvement in diverse physiological processes—TGF-β1 undeniably remains the most potent profibrotic cytokine (Abbad et al., 2025). Upon binding to its type II receptor, TGF-β1 recruits and phosphorylates the type I receptor, which subsequently phosphorylates Smad2 and Smad3. These activated Smads form a complex with Smad4 and subsequently undergo nuclear translocation to initiate the transcription of fibrotic genes (e.g., *COL1A1*, *FN1*, and *ACTA2*), a process negatively regulated by Smad7 (Meng et al., 2016). Beyond the canonical Smad pathway, TGF-β1 activates non-Smad cascades, including the MAPK and PI3K/protein kinase B (AKT) pathways, to further drive myofibroblast activation, excessive ECM synthesis, and the inhibition of ECM degradation (Meng et al., 2016). Our study confirms that AS-IV effectively suppresses both TGF-β/Smad and non-Smad signaling pathways (Song et al., 2018; Hu et al., 2022; Yu et al., 2022; Xu et al., 2025), thereby exerting its anti-fibrotic pharmacological effects. In addition, the mitigation and alleviation of renal fibrosis by AS-IV also involves inflammation-related signaling pathways [TLR/MyD88, sirtuin 1 (SIRT1)/NF-κB/NOD-like receptor protein 3 (NLRP3)/Caspase-1] (Yang et al., 2017; Yang et al., 2018; Wang et al., 2018a; Wang et al., 2019; Yuan et al., 2022; Qing et al., 2024), oxidative stress-related pathways [p62/kelch-like ECH-associated protein 1 (Keap1)/NRF2/HO-1] (Gao et al., 2021; Wang and Su, 2019), as well as the hypoxia-inducible factor-1alpha (HIF-1α) (Su and Song, 2025), cAMP/protein kinase A (PKA), and AMP-activated protein kinase (AMPK)/endothelial nitric oxide synthase (eNOS) signaling pathways (He et al., 2025; Li G. L. et al., 2023). The associated mechanisms of action are illustrated in Figure 13, and detailed mechanistic interactions are summarized in Supplementary Table S7.

### 4.3 Future perspectives

In CKD, the replacement of parenchymal tissue by ECM serves as the pathological hallmark of fibrosis, leading to irreversible structural damage. Although reversing fibrosis is considered the ultimate therapeutic goal in CKD, achieving such reversal in humans—unlike in rodent models—would likely require decades, even if biologically feasible (Ruiz-Ortega et al., 2020). Additionally, the clinical outcomes of monoclonal antibodies targeting the TGF-β signaling pathway have been largely disappointing. Specifically, in addition to demonstrating limited efficacy, direct systemic blockade frequently resulted in unacceptable adverse effects, given that TGF-β is indispensable for normal tissue homeostasis and immune regulation (Voelker et al., 2017; Vincenti et al., 2017; Deng et al., 2024; Jing et al., 2025). This clinical failure underscores that intervening in a single molecular cascade or mechanism is not only potentially toxic but also insufficient to resolve a highly complex pathology like fibrosis. Consequently, there remains a critical unmet need for therapies capable of halting CKD progression or promoting renal regeneration through safer, multi-target mechanisms.

As previously discussed, diverse pathophysiological mechanisms, including inflammation, oxidative stress, autophagy, and apoptosis, are integrally involved in fibrogenesis. Emerging evidence highlights that mitochondria, acting as key arbiters of cell fate, are deeply intertwined with these varied processes. Extensive research in cardiac, pulmonary, and hepatic fibrosis has shown that ameliorating mitochondrial dysfunction effectively mitigates fibrogenesis in these organs (Pham et al., 2024; Jiang Y. et al., 2025; Jin et al., 2026). Similarly, recent studies in CKD corroborate that restoring mitochondrial function protects renal function and halts fibrotic progression, although the precise underlying mechanisms remain incompletely elucidated (Zhao et al., 2024; Singh A. et al., 2026; Singh L. et al., 2026). Regarding AS-IV, existing reports have confirmed its ability to significantly improve mitochondrial function, suppress oxidative stress, and reduce podocyte apoptosis in mouse models of DKD, thereby preserving renal function and attenuating renal injury (Shen et al., 2023). However, direct molecular evidence definitively linking the anti-fibrotic effects of AS-IV to mitochondrial modulation in the kidney is still lacking. Therefore, elucidating the specific molecular mechanisms linking mitochondrial dysfunction to renal fibrosis and comprehensively defining the modulatory role of AS-IV within this axis represents a highly promising avenue for future research.

### 4.4 Translational challenges and future clinical trial considerations

Despite the promising renoprotective effects observed *in vivo*, translating AS-IV from preclinical models to routine clinical practice faces considerable hurdles. Currently, only one traditional Chinese medicine injection (Huangqi injection) containing AS-IV as a primary active metabolite is approved for clinical use (Xu et al., 2022). The primary translational bottleneck is its extremely low oral bioavailability (absolute bioavailability in rats: only 2.2%). To evaluate the clinical plausibility of the effective preclinical doses, we calculated the human equivalent doses using the FDA’s body surface area normalization method. In our included studies, the effective doses ranged from 1.5 to 80 mg/kg in rat models and 20–120 mg/kg in mouse models. Collectively, for a standard 60 kg adult, this translates to a daily oral requirement ranging from approximately 14.4 mg–778.2 mg of AS-IV. Achieving effects at the upper limits of this dosage spectrum presents significant practical and economic challenges in clinical settings. In addition, as a large tetracyclic triterpenoid, AS-IV exhibits low intestinal permeability and heavily relies on passive paracellular transport (Gu et al., 2004; Huang et al., 2006). Therefore, simply escalating the dose of conventional oral formulations to compensate for its poor intestinal permeability is not an optimal translational strategy. Overcoming this pharmacokinetic bottleneck relies heavily on modern pharmaceutical formulation technologies. Although recent pharmaceutical advances—such as structural modifications and novel drug delivery systems (e.g., nanocarriers)—show immense potential in bypassing these absorption barriers, these technologies require further optimization before clinical deployment (Qing et al., 2019; Jiang M. et al., 2025).

Moreover, significant species differences complicate the translational landscape. Preclinical murine models typically rely on acute, highly controlled fibrotic triggers, whereas human CKD is a highly heterogeneous, long-term pathological process influenced by metabolic and hemodynamic comorbidities. This physiological gap, coupled with the current lack of rigorous Phase I/II clinical trials, leaves the optimal therapeutic window, maximum tolerated dose, and comprehensive toxicological and pharmacokinetic profiles of AS-IV largely exploratory (Li et al., 2012; Xu et al., 2013).

Moving forward, the design of future clinical trials must carefully address these limitations. Initial trials should prioritize establishing a reliable pharmacokinetic and safety profile in humans by utilizing advanced, highly bioavailable AS-IV formulations. Additionally, future trial designs should not rely solely on late-stage clinical endpoints like serum creatinine. Given that the current diagnostic gold standard for renal fibrosis—renal biopsy—has inherent drawbacks, including delayed detection, invasiveness, and bleeding risks, sensitive early-stage biomarkers should be incorporated (Rupprecht et al., 2024; Wu et al., 2025). By identifying these early molecular signatures, researchers can better stratify patients and precisely determine the intervention strategies for AS-IV.

### 4.5 Limitations

Several limitations and considerations must be acknowledged when interpreting the findings of this systematic review: First, potential language bias may exist. Due to practical resource constraints, the literature search was restricted to English and Chinese databases. As a traditional *Qi*-invigorating botanical drug and a recognized functional food, Astragali Radix is widely utilized in the traditional medicine of East and Northeast Asia. Among the 34 included studies, the first authors of 33 studies are affiliated with Chinese institutions. Consequently, relevant studies published in Japanese, Korean, or other languages from regions where Astragali Radix is commonly used may have been inadvertently excluded, thereby introducing language bias. Second, the pooled effect estimates for specific secondary outcomes should be interpreted with caution. Several downstream molecular markers—specifically, E-cadherin, Smad7, p-Smad3, MDA, HO-1, IL-1β, and IL-6—were assessed in only a very limited number of studies (fewer than five studies for each marker). Although pooling these small datasets was methodologically feasible, the inherent lack of statistical power limited our ability to precisely estimate the true effect sizes and between-study heterogeneity. Third, the suboptimal methodological quality of the included studies compromises the overall strength and validity of our conclusions. The non-uniform purity and complex sources of AS-IV used in the included studies, combined with the SYRCLE assessment revealing poor reporting of randomization, allocation concealment, and blinding, may have led to an overestimation of the pooled effect sizes, thereby warranting a conservative interpretation of our overall findings. The lack of internal validity in preclinical models is one of the primary reasons for the unsuccessful translation of animal data into human clinical trials, severely undermining the clinical translational relevance of the results. Accordingly, we urge future animal studies to strictly adhere to the latest ARRIVE or PREPARE guidelines to improve study quality, and we look forward to incorporating newly published, high-quality *in vivo* studies in future updates to address this shortcoming and provide more robust evidence. Fourth, although subgroup analyses were conducted in an attempt to elucidate the high statistical heterogeneity observed in the primary outcomes, factors contributing to this heterogeneity beyond publication year, underlying animal disease models, intragastric dose, and treatment duration remain largely unelucidated, underscoring the need for more standardized experimental protocols. Fifth, the current literature largely fails to differentiate between primary pharmacological targets and secondary phenotypic changes. Although we synthesized a multitude of mechanisms, the vast majority—such as the attenuation of inflammation and oxidative stress, as well as the modulation of apoptosis and autophagy—likely represent downstream consequences of ameliorated overall tissue injury rather than direct drug targets. Although some studies employed specific inhibitors (e.g., the HIF-1α inhibitor LW6) to confirm certain pathways as critical downstream mediators for AS-IV’s effects, these functional rescue experiments do not equate to direct target engagement. Due to the absence of direct evidence, the definitive primary upstream receptor or direct molecular target of AS-IV remains elusive. Consequently, the mechanistic networks outlined herein should be interpreted cautiously as a constellation of downstream modulators rather than confirmed primary pharmacological targets. Sixth, a significant translational gap exists due to the rapid evolution of standard-of-care therapies in nephrology. Over half of the studies included in this review were published over 5 years ago. During this timeframe, the clinical landscape for CKD has undergone a paradigm shift with the widespread introduction of novel disease-modifying agents, including sodium-glucose cotransporter-2 inhibitors, glucagon-like peptide-1 receptor agonists, endothelin receptor antagonists, and non-steroidal mineralocorticoid receptor antagonists. Consequently, the baseline conditions of the animal models in these earlier preclinical studies do not accurately reflect the current clinical reality, where patients are typically managed with these foundational therapies. This discrepancy highlights a critical limitation in extrapolating historical preclinical effects to modern clinical applications.

## 5 Conclusion

As the first systematic review evaluating AS-IV across etiologically diverse models of renal fibrosis, our findings substantiate its promising anti-fibrotic effects. The underlying pharmacological mechanisms in animal models primarily involve ameliorating renal function, reducing proteinuria, suppressing inflammation and oxidative stress, modulating apoptosis and autophagy, and attenuating collagen deposition. However, the suboptimal methodological quality of the included studies inherently weakens the strength of this evidence; therefore, these positive findings must be interpreted with caution. Moreover, addressing the complex pharmacokinetic constraints and bridging the existing translational bottlenecks remain paramount. Moving forward, the execution of stringently designed, standardized *in vivo* investigations, coupled ultimately with well-powered, double-blind, randomized clinical trials, is necessary to rigorously validate the therapeutic utility of AS-IV against progressive human renal fibrogenesis.

## Data Availability

The original contributions presented in the study are included in the article/Supplementary Material, further inquiries can be directed to the corresponding author.

## Glossary

AKT: protein kinase B
AMPK: AMP-activated protein kinase
AS-IV: Astragaloside IV
BUN: blood urea nitrogen
cAMP: cyclic adenosine monophosphate
CAT: catalase
CKD: chronic kidney disease
DKD: diabetic kidney disease
ECM: extracellular matrix
eNOS: endothelial nitric oxide synthase
ESKD: end-stage kidney disease
GSH-Px: glutathione peroxidase
HIF-1α: hypoxia-inducible factor-1alpha
HO-1: heme oxygenase-1
ICAM-1: intercellular adhesion molecule 1
IL-1β: interleukin-1 beta
IL-6: interleukin-6
IRI: ischemia-reperfusion injury
Keap1: kelch-like ECH-associated protein 1
LC3: microtubule-associated protein light chain 3
MDA: malondialdehyde
mTOR: mammalian target of rapamycin
MyD88: myeloid differentiation factor 88
NF-κB: nuclear factor κB
NLRP3: nod-like receptor protein 3
NRF2: nuclear factor E2-related factor 2
pEMT: partial epithelial-mesenchymal transition
PMT: pericyte-myofibroblast transition
PKA: protein kinase A
p-Smad3: phosphorylated Smad3
ROS: reactive oxygen species
RRT: renal replacement therapies
Scr: serum creatinine
SIRT1: sirtuin 1
SOD: superoxide dismutase
TGF-β: transforming growth factor-beta
TLR: toll-like receptor
TNF-α: tumor necrosis factor-alpha
UACR: urinary albumin to creatinine ratio
UUO: unilateral ureteral obstruction
VCAM-1: vascular cell adhesion molecule 1
α-SMA: α-smooth muscle actin.
