# Supplementary material for: Effects and potential mechanisms of astragaloside IV in animal models of renal fibrosis: a systematic review and meta-analysis
Source: Front Pharmacol. 2026 Aug 25;17:1867891. doi: 10.3389/fphar.2026.1867891 (PMC13550134; doi:10.3389/fphar.2026.1867891)

Supplementary Materials

Project	Content	Page
Table S1	Summary of primary outcomes	2
Table S2	Basic characteristics of the 34 included studies	3-14
Table S3	Information on AS-IV used	15
Table S4	Subgroup analysis of AS-IV	16-18
Table S5	Egger's publication bias	19
Table S6	Egger's test and trim-and-fill analysis	20
Table S7	Mechanisms of the anti-fibrosis effect of AS-IV	21-22
Figure S1	Linear dose-response analysis charts	23
Figure S2	Non-linear dose-response analysis charts	24
Figure S3	Forest plot: effect of AS-IV on blood glucose	25

Supplementary Table S1. Summary of primary outcomes.

No.	Primary outcomes
**1**	Serum creatinine
**2**	Blood urea nitrogen
**3**	Proteinuria
**4**	Transforming growth factor-beta 1
**5**	Fibronectin
**6**	Alpha-smooth muscle actin

Supplementary Table S2. Basic characteristics of the 34 included studies

Study (Year)	Species (Sex; n=treatment/control group); Weight; Age	Model	Treatment group (AS-IV) (administration; course of treatment)	Control group	Kidney histopathology	Outcomes
Yu et al. 2004	Sprague-Dawley rats (male; 6/6); 200-220; -	unilateral renal artery clamping- induced ischemia-reperfusion injury model	By Intragastric; normal saline and 0.1 g/L 2 mL; 6 days	By Intragastric; same amount of normal saline; 6 days	H&E staining; Sirius Red staining	1. UP<br>2. Scr<br>3. TGF-β1
Zhu et al. 2014	Sprague-Dawley rats (male; 9/9); 200-240; -	unilateral ureteral obstruction model	By Intragastric; 1.5 mg/kg/d; 14 days	By Intragastric; same amount of distilled water; 14 days	-	1. Scr<br>2. BUN<br>3. Fibronectin<br>4. CTGF<br>5. TGF-β
Wang et al. 2014	Sprague-Dawley rats (male; 15/15); 200-250; -	unilateral ureteral obstruction model	By Intragastric; 3.33 、 10 、 33 mg/kg/d(dissolved in 0.5% CMC-NA solution); 14 days	-	H&E staining; Masson’s trichrome staining	1. Scr<br>2. BUN<br>3. TGF-β1<br>4. CTGF<br>5. α-SMA<br>6. Fibronectin<br>7. Col1, 3<br>8. p-Smad2, 3<br>9. Smad7
Xu et al. 2014a	C56BL/6 mice (male; 10/10); 18-22; 8 weeks	unilateral ureteral obstruction model	By Intragastric; 20 mg/kg/d; 7 days	By Intragastric; same amount of solvent; 7 days	Masson's trichrome staining	1. Kidney index<br>2. TGF-β1<br>3. Fibronectin<br>4. Col4<br>5. α-SMA<br>6. p-p38

Xu et al. 2014b	C56BL/6 mice (male; 10/10); 16-18; 6-8 weeks	unilateral ureteral obstruction model	Intraperitoneal injection; 20 mg/kg/d; 14 days	Intraperitoneal injection; same amount of vehicle; 14 days	H&E staining; Masson's trichrome staining	1. Kidney index<br>2. Scr<br>3. BUN<br>4. Col4<br>5. Fibronectin<br>6. TGF-β1<br>7. α-SMA<br>8. Cleaved-Caspase 3<br>9. p-p38/p-38<br>10. p-JNK/JNK<br>11. P-Erk1/2/Erk1/2
Chen et al. 2016	Sprague-Dawley rats (male; 10/10); 180-200; 4-6 weeks	STZ- induced DKD model	By Intragastric; normal saline and 1mmol/L 5 mL; 30 days	By Intragastric; normal saline 5 mL qd; 30 days	H&E staining; Periodic Acid-Schiff staining	1. 24 h UP<br>2. Scr<br>3. Blood glucose
Guo et al. 2016	db/db mice (male; 10/10); -; 8 weeks	db/db type 2 diabetes mellitus model	By Intragastric; 2 、 6 、 18 mg/kg/d(dissolved in 0.5% CMC-Na solution); 8 weeks	By Intragastric; same amount of 0.5% CMC-Na solution; 8 weeks	Periodic Acid-Schiff staining	1. BUN<br>2. Scr<br>3. HbA1c<br>4. MCP-1<br>5. TNF-α<br>6. Kidney index<br>8. Podocin<br>9. Nephrin<br>10. p-PERK/PERK 、 p-eIF2α/eIF2α、p-IRE1α/ IRE1α、p-JNK/JMK、CHOP<br>11. Cleaved-Caspase 3、9、12

						etc.
Wang et al. 2017	KKAy mice (male; 10/10); 22-24; 14 weeks	KKAy type 2 diabetes mellitus model	By Intragastric; 40 mg/kg/d; 10 weeks	Drink water; 10weeks	H&E staining; Masson's trichrome staining	1. FBG<br>2. TGF-β1<br>3. Smad2/3<br>4. α-SMA
Yang et al. 2017	C56BL/6 mice (-; 10/10); 15-25; -	unilateral ureteral obstruction model	By Intragastric; 10、30、50 mg/kg/d qd; 14 days	By Intragastric; same amount of normal saline; 14 days	H&E staining; Masson's trichrome staining	1. Scr<br>2. BUN<br>3. α-SMA<br>4. Fibronectin<br>5. TLR2, 4<br>6. My D88<br>7. TRAF-6<br>8. NF-κB<br>9. TNF-α<br>10. IL-6
Fan et al. 2017	Wistar rats (male; 8/11); 150-168; 4 weeks	STZ- induced DKD model	By Intragastric; 10 mg/kg/d(dissolved in 1% CMC-NA solution); 6 weeks	-	-	1. Blood glucose<br>2. Kidney index<br>3. UACR<br>4. Scr<br>5. BP<br>6. TGF-β<br>7. Col4<br>8. AGT, renin<br>9. gp91-phox, p22-phox
Zhou et al. 2017	C56BL/6 mice (male; 5/5); 18-24; 8 weeks	unilateral ureteral obstruction model	By Intragastric; 20、40 mg/kg/d; 1 week	By Intragastric; same amount of normal saline; 1 week	-	1. Col1<br>2. Fibronectin

						3. CD68, CD3<br>4. TNF-α<br>5. IL-1β<br>6. TLR4<br>7. p65<br>8. IκBα
Yang et al. 2018	C56BL/6 mice (-; 10/10); 15-25; -	unilateral renal artery clamping- induced ischemia-reperfusion injury model	By Intragastric; 30 mg/kg/d; 30 days	By Intragastric; 0.1% ethanol solution in double-distilled water of the same amount; 30 days	H&E staining; Masson's trichrome staining	1. Scr<br>2. BUN<br>3. α-SMA<br>4. Fibronectin<br>5. TLR4<br>6. My D88<br>7. TRAF-6<br>8. INF-γ<br>9. TNF-α<br>10. IL-6<br>11. NF-Kb<br>etc.
Song et al. 2018	C56BL/6 mice (male; 6/6); 16-18; 5-6 weeks	STZ- induced DKD model	Orally; 5 g/kg standard chow; 8 weeks	Orally; standard chow; 8 weeks	Periodic Acid-Schiff staining	1. 24 h UP<br>2. ACR<br>3. Blood glucose<br>4. Scr<br>5. BUN<br>6. TG, HDL-C, LDL-C, ALT, AST, Alb, TP<br>7. Kidney index

						8. NGAL, NAG<br>9. TGF-β1<br>10. p-MEK1/2/MEK1/2<br>11. p-ERK1/2/ERK1/2<br>12. p-RSK2/RSK2
Wang et al. 2018a	KKAy mice (male; 12/12); -; 8 weeks	KKAy type 2 diabetes mellitus model	By Intragastric; 40 mg/kg/d; 12 weeks	By Intragastric; same amount of distillate; 12 weeks	H&E staining; Masson's trichrome staining	1. Beclin1<br>2. LC3 II<br>3. ACR<br>4. UP
Wang et al. 2018b	KKAy mice (male; 12/12); -; 8 weeks	KKAy type 2 diabetes mellitus model	By Intragastric; 40 mg/kg/d(dissolved in 1% CMC-Na solution); 12 weeks	By Intragastric; distillate; 12 weeks	H&E staining; Masson's trichrome staining; Periodic Acid-Schiff staining	1. miR-21<br>2. UACR<br>3. UP
Mao et al. 2019	Sprague-Dawley rats (female/male; 6/6); 200-220; 9 weeks	STZ- induced DKD model	By Intragastric; 40 mg/kg/d; 8 weeks	Orally; same amount of normal saline; 8 weeks	H&E staining; Masson's trichrome staining	1. Kidney index<br>2. Blood glucose<br>3. UP<br>4. Scr<br>5. BUN<br>6. miR-192<br>7. TGF-β1<br>8. Smad3, 7<br>9. Col1<br>10. α-SMA
Wang et al. 2019a	Sprague-Dawley rats (male; 12/12); 160-208; -	STZ- induced DKD model	By Intragastric; 20, 40, 80 mg/kg/d; 8 weeks	By Intragastric; same amount of normal saline qd; 8 weeks	H&E staining; Periodic Acid-Schiff staining	1. Kidney index<br>2. Blood glucose<br>3. 24 h UP<br>4. Scr

						5. BUN<br>6. MDA<br>7. SOD<br>8. GSH-Px<br>9. TGF-β1<br>10. Nrf2<br>11. HO-1
Wang et al. 2019b	KKAy mice (male; 12/12); -; 8 weeks	KKAy type 2 diabetes mellitus model	By Intragastric; 40 mg/kg/d; 12 weeks	By Intragastric; distillate; 12 weeks	H&E staining; Masson's trichrome staining; Periodic Acid-Schiff staining, EM	1. UP<br>2. UACR<br>3. Col4<br>4. Fibronectin<br>5. TGF-β<br>6. E-cadherin<br>7. N-cadherin
Zhang et al. 2020	Sprague-Dawley rats (male; 8/8); -; 4 weeks	STZ- induced DKD model	By Intragastric; 80 mg/kg/d(dissolved in 0.5% CMC-Na solution); 12 weeks	By Intragastric; 0.5% CMC-Na solution; 12 weeks	H&E staining; Masson's trichrome staining; Periodic Acid-Schiff staining, EM	1. Kidney index<br>2. 24 h UP<br>3. Scr<br>4. BUN<br>5. Ccr<br>6. NOD2, JUN, PANX1, NOX2, TXNIP, Caspase1, TRX1<br>7. AGEs<br>8. IL-1β<br>9. IL-18<br>10. Col4<br>11. Fibronectin

Zhou et al. 2021	C56BL/6 mice (male; 10/10); 18.1-22.1; 8 weeks	unilateral ureteral obstruction model	By Intragastric; 20 mg/kg/d; 2 weeks	By Intragastric; same amount of normal saline; 2 weeks	Masson's trichrome staining	1. TGF-β1
Gao et al. 2021	C56BL/6 mice (male; 8/8); -; 8 weeks	tacrolimus-induced chronic nephrotoxicity model	By Intragastric; 10、20、40 mg/kg/d; 4 weeks	-	H&E staining; Masson's trichrome staining	1. Scr<br>2. BUN<br>3. TGF-β1<br>4. Col1<br>5. α-SMA<br>6. MDA<br>7. SOD<br>8. CAT<br>9. GSH-Px<br>10. Nrf2<br>11. NQO1, GCLC<br>12. HO-1<br>13. Keap1, p-p62
Jiang et al. 2022	Sprague-Dawley rats (male; 15/14); 180-200; 7 weeks	5/6 nephrectomized model	By Intragastric; 50 mg/kg/d(dissolved in saline) qd; 8 weeks	By Intragastric; normal saline qd; 8 weeks	H&E staining; Masson's trichrome staining	1. 24 h UP<br>2. Scr<br>3. BUN<br>4. α-SMA<br>5. TGF-β1<br>6. p-Smad3<br>7. Smad7
Yuan et al. 2022	Sprague-Dawley rats (male; 8/8); 220-260; -	STZ- induced DKD model	By Intragastric; 10 mg/kg/d; 8 weeks	-	H&E staining; Masson's trichrome staining	1. Serum alb<br>2. TP<br>3. Scr<br>4. BUN

						5. NLRP3<br>6. caspase-1<br>7. TGF-β
Zhang et al. 2022	Wistar rats (female/male; 10/10); 60-80; 30 days	PTU- induced hypothyroidism model	By Intragastric; 20、40 mg/kg/d; 5 weeks	By Intragastric; same amount of normal saline; 5 weeks	H&E staining; Masson's trichrome staining	1. FT3, 4, TSH<br>2. Kidney index<br>3. 24 h UP<br>4. Scr<br>5. BUN<br>6. TGF-β1<br>7. TβRI<br>8. p-Smad3/ Smad3<br>9. Smad7<br>10. α-SMA<br>11. Col1
Hu et al. 2022	db/db mice (male; 10/10); -; 8 weeks	db/db type 2 diabetes mellitus model	By Intragastric; 10 、 20 、 40 mg/kg/d(dissolved in CMC-Na solution); 12 weeks	By Intragastric; same amount of CMC-Na solution; 12 weeks	H&E staining; Masson's trichrome staining; Periodic Acid-Schiff staining; Periodic acid-silver methenamine staining	1. Kidney index<br>2. FBG<br>3. Scr<br>4. BUN<br>5. 24 h UP<br>6. ALT, AST<br>7. TG, TC<br>8. E-cadherin<br>9. Vimentin<br>10. α-SMA<br>11. IL-1β<br>12. CX3CL1

						13. p-c-Raf/c-Raf<br>14. p-MEK/MEK<br>15. p-ERK/ERK
Yu et al. 2022	Sprague-Dawley rats (-; 8/8); 180-220; -	unilateral renal artery clamping- induced ischemia-reperfusion injury model	By Intragastric; 80 mg/kg/d; 14 days	-	H&E staining; Masson's trichrome staining	1. Scr<br>2. BUN<br>3. α-SMA<br>4. Col1<br>5. Fibronectin<br>6. p-Akt<br>7. p-GSK3β/GSK3β
Tang et al. 2022	C56BL/6 mice (male; 12/12); -; 6-8 weeks	unilateral renal artery clamping- induced ischemia-reperfusion injury model	By Intragastric; 20 mg/kg/d; 7 days	By Intragastric; same amount of normal saline; 7 days	H&E staining; Masson's trichrome staining	1. Fibronectin<br>2. Col1<br>3. α-SMA
Li et al. 2023	Sprague-Dawley rats (male; 10/10); 180-200; 8 weeks	adenine- induced chronic kidney disease model	By Intragastric; 40 、 80 mg/kg/d(dissolved in 0.5% CMC-Na solution); 4 weeks	By Intragastric; same amount of 0.5% CMC-Na solution; 4 weeks	H&E staining; Masson's trichrome staining; Sirius Red staining	1. Kidney index<br>2. Scr<br>3. BUN<br>4. Ccr<br>5. Fibronectin<br>6. Vimentin<br>7. α-SMA<br>8. E-cadherin<br>10. ALDH2<br>11. p-Akt/Akt<br>12. p-mTOR/ mTOR<br>13. ATG7<br>14. Beclin1

						15. LC3 II/LC3 I<br>etc.
Li et al. 2023	Sprague-Dawley rats (male; 9/9); 160-180; 5 weeks	STZ- induced DKD model	By Intragastric; 40 mg/kg/d(dissolved in 1% CMC-NA solution); 12 weeks	By Intragastric; same amount of normal saline; 12 weeks	H&E staining; Masson's trichrome staining; Periodic Acid-Schiff staining	1. FBG<br>2. Scr<br>3. BUN<br>4. UACR<br>5. p-AMPK/AMPK<br>6. eNOS
Arda et al. 2024	Wistar rats (female; 10/10); 200-210; 30 days	cisplatin-induced kidney injury model	Intraperitoneal injection; 80 mg/kg/d; 4 weeks	Intraperitoneal injection; normal saline 1 mL/kg/d; 4 weeks	H&E staining	1. MDA<br>2. TNF-α<br>3. KIM-1<br>4. NGAL<br>5. Nephrin<br>6. Scr<br>7. BUN<br>8. TGF-β1
Qing et al. 2024	MRL/lpr mice(female; 8/8); 20-22; 7 weeks	Systemic Lupus Erythematosus Model	By Intragastric; 120 mg/kg/d(dissolved in distillate); 4 weeks	By Intragastric; same amount of normal saline; 4 weeks	H&E staining; Masson's trichrome staining	1. Kidney index<br>2. Scr<br>3. BUN<br>4. 24 h UP<br>5. IL-1β<br>6. IL-18<br>7. p-p65/p65<br>8. p-IκBα/IκBα<br>9. NLRP3<br>10. cleaved caspase-1/pro caspase-1

						etc.
Xu et al. 2025	C56BL/6J mice (male; 10/10); 18-22; 7 weeks	unilateral renal artery clamping- induced ischemia-reperfusion injury model	By Intragastric; 40、80 mg/kg/d; 14 days	By Intragastric; 10 mL/kg/d; 14 days	H&E staining; Masson's trichrome staining; Periodic Acid-Schiff staining; Sirius Red staining	1. Kidney index<br>2. Scr<br>3. BUN<br>4. Col1<br>5. α-SMA<br>6. TGF-β1<br>7. TPL2<br>8. CTGF
Su et al. 2025	C56BL/6N mice (male; 10/10); 20-25; 8 weeks	STZ- induced DKD model	By Intragastric; 100 mg/kg/d; 4 weeks	By Intragastric; same amount of normal saline; 4 weeks	H&E staining; Masson's trichrome staining; Periodic Acid-Schiff staining	1. FBG<br>2. UACR<br>3. Scr<br>4. Col1<br>5. α-SMA<br>6. TNF-α<br>7. IL-6<br>8. IL-10<br>9. Arg-1<br>10. p-p65/p65<br>11. HIF-1α
He et al. 2025	Sprague-Dawley rats (male; 6/6); 160-180;-	unilateral ureteral obstruction model	By Intragastric; 40 mg/kg/d; 14 days	By Intragastric; normal saline 5 mL/kg/d; 14 days	H&E staining; Masson's trichrome staining	1. Scr<br>2. BUN<br>3. α-SMA<br>4. Fibronectin<br>5. Col1<br>6. cAMP

	7. PKA
	8. GPX4
	9. HO-1
	10. xCT

Alb: albumin; AGT: angiotensinogen; AKT: protein kinase B; Alb: albumin; ALDH2: aldehyde dehydrogenase 2; ALT: alanine aminotransferase; AMPK: AMP-activated protein kinase; Arg-1: arginase-1; AS-IV: Astragaloside IV; AST: aspartate aminotransferase; ATG7: autophagy-related gene 7; BP: blood pressure; BUN: blood urea nitrogen; cAMP: cyclic adenosine monophosphate; CAT: catalase; Ccr: endogenous creatinine clearance rate; CHOP: C/EBP homologous protein; Col I: type I collagen; Col IV: type IV collagen; CTGF: connective tissue growth factor; CX3CL1: C-X3-C motif chemokine ligand 1; eIF2α: eukaryotic initiation factor 2 subunit α; EM: electron microscope; eNOS: endothelial nitric oxide synthase; ERK: extracellular signal-regulated kinase; FBG: fasting blood glucose; FT3: free triiodothyronine; FT4: free thyroxine; GCLC: glutamate-cysteine ligase catalytic subunit; GPX4: glutathione peroxidase 4; GSH-Px: glutathione peroxidase; GSK3β: glycogen synthase kinase-3β; HbA1c: hemoglobin A1c; HDL-C: high-density lipoprotein cholesterol; HIF-1α: hypoxia-inducible factor-1alpha; HO-1: heme oxygenase 1; ICAM-1: intercellular adhesion molecule 1; IL-1β: interleukin-1 beta; IL-6: interleukin-6; INF-γ: interferon-γ; IRE1α: inositol-requiring enzyme 1α; IκBα: NF-κB inhibitor α; JNK: c-Jun N-terminal Kinase; JUN: jun proto-oncogene; Keap1: kelch-like ECH-associated protein 1; KIM-1: kidney injury molecule-1; LC3: microtubule-associated protein light chain 3; LDL-C: low-density lipoprotein cholesterol; MCP-1: monocyte chemoattractant protein-1; MDA: malondialdehyde; MEK: mitogen-activated protein kinase; mTOR: mammalian target of rapamycin; My D88: myeloid differentiation factor 88; NAG: neutrophil gelatinase-associated lipocalin; NF-κB: nuclear factor Kb; NGAL: urinary neutrophil gelatinase-associated lipocalin; NLRP3: nod-like receptor protein 3; NOD2: nucleotide-binding oligomerization domain 2; NOX2: NADPH oxidases 2; NQO1: NAD(P)H:quinone oxidoreductase 1; NRF2: nuclear factor E2-related factor 2; PANX1: pannexin1; PERK: protein kinase R-like ER kinase; PKA: protein kinase A; p-Smad3: phosphorylated Smad3; RSK2: ribosomal S6 kinase 2; Scr: serum creatinine; SIRT1: sirtuin 1; SOD: superoxide dismutase; STZ: streptozocin; TC: total cholesterol; TG: triglyceride; TGF-β: transforming growth factor-beta; TLR: toll-like receptor; TNF-α: tumor necrosis factor-alpha; TP: total protein; TPL2: tumor progression locus 2; TRAF-6: TNF receptor associated factor 6; TSH: thyroid-stimulating hormone; TXNIP: thioredoxin-interacting protein; TβRI: transforming growth factor-β receptor type I; UACR: urinary albumin to creatinine ratio; UP: urinary protein; VCAM-1: vascular cell adhesion molecule 1; xCT: solute carrier family 7 member 11; α-SMA: α-smooth muscle actin

Supplementary Table S3. Information on the AS-IV used in each study.

Study, year	Source	Purity	Quality control reported
Yu et al. 2004	Shanghai Drug Control, Shanghai, China	83.73%	Unknown
Zhu et al. 2014	Winherb Medical Science, Shanghai, China	Unknown	Lot: 110702
Xu et al. 2014a	National Institutes for Food and Drug Control, Beijing, China	>98%	Unknown
Wang et al. 2014	National Institutes for Food and Drug Control, Shanghai, China	98%	HPLC
Xu et al. 2014b	PureOne Biotechnology, Shanghai, China	>98%	Unknown
Chen et al. 2016	Ronbio Scientific, Shanghai, China	Unknown	Lot: 14051502
Guo et al. 2016	Bogoo Biotechnology, Shanghai, China	98%	Unknown
Wang et al. 2017	Sigma–Aldrich, MO, USA	Unknown	Unknown
Yang et al. 2017	Must Bio-Technology, Chengdu, China	98%	Lot: 110702
Fan et al. 2017	Winherb Medical Science, Shanghai, China	≥98%	HPLC
Zhou et al. 2017	Conbon Bio-tech, Chengdu, China	Unknown	Unknown
Yang et al. 2018	Must Bio-Technology, Chengdu, China	Unknown	Lot: 110702
Wang et al. 2018a	Sigma-Aldrich, MO, USA	≥98%	HPLC
Wang et al. 2018b	Sigma-Aldrich, MO, USA	≥98%	HPLC
Song et al. 2018	Unknown	Unknown	Unknown
Wang et al. 2019a	Cogon Biotechnology, Chengdu, China	>80%	Lot: ZLI20305
Mao et al. 2019	Jizhi Biochemical Technology, Shanghai, China	≥99%	HPLC
Wang et al. 2019b	Sigma–Aldrich, MO, USA	≥98%	HPLC
Zhang et al. 2020	Dilger Medical Technology, Nanjing, China	>98%	Unknown
Zhou et al. 2021	National Institutes for Food and Drug Control, Beijing, China	>98%	Unknown
Gao et al. 2021	Jintaihe Pharmaceutical Chemical Technology, Chengdu, China	≥98%	Lot: KT201901
Jiang et al. 2022	National Institutes for Food and Drug Control, Beijing, China	97.40%	Unknown
Yuan et al. 2022	Unknown	Unknown	Unknown
Zhang et al. 2022	Herbpurify, Chengdu, China	>98%	Unknown
Hu et al. 2022	Unknown	Unknown	Unknown
Yu et al. 2022	Mansite, Chengdu, China	≥98%	Lot: DSTDH001501
Tang et al. 2022	Sigma-Aldrich, MO, USA	Unknown	Unknown
Li et al. 2023b	Spring and Autumn Bio., Nanjing, China	≥99%	Lot: A800922
Li et al. 2023a	Jingzhu Biotechnology, Nanjing, China	≥98%	Unknown
Arda et al. 2024	Unknown	Unknown	Unknown
Qing et al. 2024	ChemFaces, Wuhan, China	≥98%	Lot: CFN99171
Xu et al. 2025	Herbpurify, Chengdu, China	98%	Lot: 88687-43-4
Su et al. 2025	Yuanye Bio-Technology, Shanghai, China	Unknown	Lot: YY20230328
He et al. 2025	MedChemExpress, Shanghai, China	Unknown	Cat: HY-N0431

Supplementary Table S4. The subgroup analysis of AS-IV on Scr, BUN, proteinuria, TGF-β1, fibronectin, and α-SMA

Project	Subgroup	Parameter	No. of studies	SMD [95% CI]	I^2	Heterogeneity
Scr	Year	pre-2020	12	-1.71[-2.52, -0.9]	85%	I^2 = 60% P = 0.11
		2020 and later	14	-3.01[-4.39, -1.62]	92%	
	Animal model	DKD	11	-1.13[-2.18, -0.08]	90%	I^2 = 27.7% P = 0.25
		UUO	5	-3.9[-5.46, -0.72]	95%	
		IRI	3	-2.46[-4.86, -0.06]	91%	
	Dose	≤ 30 mg/kg	17	-1.39[-2.19, -0.59]	82%	I^2 = 74.2% P = 0.02
		> 30 and ≤ 60 mg/kg	12	-3.73[-5.18, -2.29]	88%	
		> 60 mg/kg	7	-2.18[-4.24, -0.11]	92%	
	Duration	≥6 w	10	-2.24[-3.44, -1.04]	89%	I^2 = 0% P = 0.76
		< 6 w	16	-2.49[-3.5, -1.48]	90%	

Project	Subgroup	Parameter	No. of studies	SMD [95% CI]	I^2	Heterogeneity
BUN	Year	pre-2020	9	-1.52[-2.64, -0.4]	87%	I^2 = 87.4% P = 0.005
		2020 and later	13	-4.3[-5.88, -2.72]	91%	
	Animal model	DKD	8	-2.15[-3.56, -0.73]	89%	I^2 = 0% P = 0.73
		UUO	5	-1.64[-3.24, -0.04]	90%	
		IRI	2	-4.34[-11.73, 3.04]	96%	
	Dose	≤ 30 mg/kg	16	-1.80[-2.68, -0.91]	82%	I^2 = 66.8% P = 0.05
		> 30 and ≤ 60 mg/kg	12	-3.32[-4.90, -1.74]	90%	
		> 60 mg/kg	6	-3.96[-5.73, -2.19]	82%	
	Duration	≥6 w	9	-2.73[-4.3, -1.16]	91%	I^2 = 0% P = 0.52
		< 6 w	13	-3.41[-4.71, -2.11]	91%	

Project	Subgroup	Parameter	No. of studies	SMD [95% CI]	I^2	Heterogeneity
Proteinuria	Year	pre-2020	7	-2.50[-3.42, -1.57]	67%	I^2 = 0% P = 0.49
		2020 and later	4	-3.28[-5.32, -1.25]	86%	
	Animal model	DKD	8	-2.08[-2.81, -1.34]	64%	-
		UUO	0	-	-	
		IRI	1	-3.3[-5.26, -1.33]	-	
	Dose	≤ 30 mg/kg	5	-1.57[-2.38, -0.77]	43%	I^2 = 55.7% P = 0.10
		> 30 and ≤ 60 mg/kg3	5	-3.14[-4.72, -1.57]	77%	

		> 60 mg/kg	3	-3.76[-6.68, -0.85]	87%	
	Duration	≥6 w	7	-2.1[-2.95, -1.24]	68%	I^2 = 68.3% P = 0.08
		< 6 w	4	-4.04[-6.02, -2.07]	78%	

Project	Subgroup	Parameter	No. of studies	SMD [95% CI]	I^2	Heterogeneity
TGF-β1	Year	pre-2020	8	-5.88[-8.14, -3.62]	92%	I^2 = 0% P = 0.88
		2020 and later	6	-6.13[-8.52, -3.75]	88%	
	Animal model	DKD	4	-2.94[-4.98, -0.91]	86%	I^2 = 56.1% P = 0.1
		UUO	4	-10.44[-17.49, -3.39]	95%	
		IRI	2	-6.69[-14.93, 1.55]	94%	
	Dose	≤ 30 mg/kg	8	-3.60[-5.09, -2.11]	82%	I^2 = 48.1% P = 0.15
		> 30 and ≤ 60 mg/kg	8	-5.67[-7.33, -4.01]	74%	
		> 60 mg/kg	2	-7.57[-15.50, 0.37]	88%	
	Duration	≥6 w	5	-3.55[-5.6, -1.5]	88%	I^2 = 85.5% P = 0.009
		< 6 w	9	-7.81[-10.24, -5.38]	92%	

Project	Subgroup	Parameter	No. of studies	SMD [95% CI]	I^2	Heterogeneity
Fibronectin	Year	pre-2020	8	-6.05[-7.73, -4.37]	78%	I^2 = 0% P = 0.62
		2020 and later	4	-5.13[-8.29, -1.97]	89%	
	Animal model	DKD	2	-3.9[-8.18, 0.39]	92%	I^2 = 62.4% P = 0.07
		UUO	7	-6.25[-8.21, -4.29]	77%	
		IRI	1	-3.38[-4.84, -1.92]	-	
	Dose	≤ 30 mg/kg	8	-4.12[-5.76, -2.49]	80%	I^2 = 28.9% P = 0.25
		> 30 and ≤ 60 mg/kg	6	-6.50[-8.75, -4.24]	71%	
		> 60 mg/kg	3	-5.33[-9.43, -1.24]	90%	
	Duration	≥6 w	2	-3.9[-8.18, 0.39]	92%	I^2 = 0% P = 0.33
		< 6 w	10	-6.18[-7.91, -4.45]	82%	

Project	Subgroup	Parameter	No. of studies	SMD [95% CI]	I^2	Heterogeneity
α-SMA	Year	pre-2020	7	-6.05[-8, -4.11]	81%	I^2 = 0% P = 0.89
		2020 and later	9	-5.86[-7.64, -4.08]	84%	
	Animal model	DKD	4	-3.88[-5.55, -2.21]	72%	I^2 = 75.1% P = 0.02
		UUO	5	-8.4[-11.34, -5.46]	79%	
		IRI	2	-9.92[-18.7, -1.14]	90%	
	Dose	≤ 30 mg/kg	11	-5.06[-6.72, -3.40]	83%	I^2 = 0%

		> 30 and ≤ 60 mg/kg	11	-4.95[-6.33, -3.58]	76%	P = 0.72
		> 60 mg/kg	4	-6.16[-8.78, -3.55]	74%	
	**Duration**	≥6 w	4	-3.84[-5.46, -2.23]	74%	I^2 = 84.6%
		< 6 w	12	-6.82[-8.44, -5.2]	81%	P = 0.01

Supplementary Table S5. Egger's publication bias of Scr, BUN, proteinuria, TGF-β1, fibronectin, and α-SMA

Standard effect		Coefficient	Standard error	t	P-value	95% Confidence interval
**Scr**	Slope	2.723772	0.7261044	3.75	0.001	1.225166 to 4.222377
	Bias	-7.266147	1.196896	-6.07	< 0.001	-9.736418 to -4.795876

Standard effect		Coefficient	Standard error	t	P-value	95% Confidence interval
**BUN**	Slope	3.77706	0.6637219	5.69	< 0.001	2.39256 to 5.16156
	Bias	-8.784075	0.9721369	-9.04	< 0.001	-10.81192 to -6.756233

Standard effect		Coefficient	Standard error	t	P-value	95% Confidence interval
**Protei nuria**	Slope	1.195397	0.6368263	1.88	0.093	-.2452038 to 2.635999
	Bias	-5.485484	0.9439668	-5.81	< 0.001	-7.620885 to -3.350083

Standard effect		Coefficient	Standard error	t	P-value	95% Confidence interval
**TGF-β1**	Slope	1.234104	0.7197792	1.71	0.112	-.3341606 to 2.802368
	Bias	-6.424607	0.8504158	-7.55	< 0.001	-8.277504 to -4.571711

Standard effect		Coefficient	Standard error	t	P-value	95% Confidence interval
**fibron ectin**	Slope	0.8726732	0.7343322	1.19	0.262	-0.7635209 to 2.508867
	Bias	-5.834214	0.7483156	-7.80	< 0.001	-7.501565 to -4.166863

Standard effect		Coefficient	Standard error	t	P-value	95% Confidence interval
**α-SMA**	Slope	0.5134023	0.5421196	0.95	0.360	-0.6493286 to 1.676133
	Bias	-5.707987	0.5442798	-10.49	< 0.001	-6.875351 to -4.540623

Supplementary Table S6. Egger's test and trim-and-fill analysis for Scr, BUN, proteinuria, TGF-β1, fibronectin, and α-SMA

Item		No. studies	Hedges's g	95% Confidence interval
Scr	Before trim and fill	26	-2.606	-3.688 to -1.524
	After trim and fill	26	-2.606	-3.688 to -1.524

Item		No. studies	Hedges's g	95% Confidence interval
BUN	Before trim and fill	22	-3.320	-4.593 to -2.046
	After trim and fill	22	-3.320	-4.593 to -2.046

Item		No. studies	Hedges's g	95% Confidence interval
Proteinuria	Before trim and fill	11	-2.993	-4.167 to -1.819
	After trim and fill	11	-2.993	-4.167 to -1.819

Item		No. studies	Hedges's g	95% Confidence interval
TGF-β1	Before trim and fill	14	-6.467	-8.843 to -4.090
	After trim and fill	15	-5.931	-8.460 to -3.402

Item		No. studies	Hedges's g	95% Confidence interval
fibronectin	Before trim and fill	12	-6.510	-9.123 to -3.897
	After trim and fill	12	-6.510	-9.123 to -3.897

Item		No. studies	Hedges's g	95% Confidence interval
α-SMA	Before trim and fill	16	-6.485	-8.292 to -4.678
	After trim and fill	16	-6.485	-8.292 to -4.678

Supplementary Table S7. Mechanisms of the anti-fibrosis effect of AS-IV

Study (Year)	The mechanisms involved
Yu et al. 2004	TGF-β1 signaling pathway ↓
Zhu et al. 2014	TGF-β/CTGF signaling pathway ↓
Wang et al. 2014	Regulating the TGF-β1/Smads signaling pathway
Xu et al. 2014a	1. TGF-β1 ↓
	2. p-p38 ↓
Xu et al. 2014b	Anti-apoptosis
Chen et al. 2016	Anti-fibrosis
Guo et al. 2016	1. Anti-endoplasmic reticulum stress
	2. Anti-podocyte apoptosis
Wang et al. 2017	Regulating TGF-β/Smads signaling pathway
Yang et al. 2017	Toll/MyD88 signaling pathway ↓
Fan et al. 2017	1. Regulating glucose tolerance
	2. Antioxidant
Zhou et al. 2017	TLR4/NF-κB signaling pathway ↓
Yang et al. 2018	Toll/MyD88 signaling pathway ↓
Song et al. 2018	MEK1/2/ERK1/2/RSK2 signaling pathway ↓
Wang et al. 2018a	1. Regulating SIRT1/NF-κB signaling pathway
	2. Induction of autophagy
	3. Inhibiting mesangial cell activation
Wang et al. 2018b	1. miR-21 ↓
	2. Inhibiting mesangial cell activation
	3. Inhibiting podocyte dedifferentiation
Mao et al. 2019	Regulating TGF-β1/Smads/miR-192 signaling pathway
Wang et al. 2019a	1. NRF2/HO-1 signaling pathway ↑
	2. Antioxidant
	3. TGF-β1 signaling pathway ↓
Wang et al. 2019b	1. Regulating SIRT1/NF-κB signaling pathway
	2. Induction of autophagy
	3. Inhibiting podocyte epithelial-mesenchymal transition
Zhang et al. 2020	Anti-inflammation
Zhou et al. 2021	TGF-β1 signaling pathway ↓
Gao et al. 2021	1. Regulating p62/Keap1/NRF2 signaling pathway
	2. Antioxidant
Jiang et al. 2022	Regulating the TGF-β1/Smads signaling pathway
Yuan et al. 2022	NLRP3/Caspase-1 signaling pathway ↓
Zhang et al. 2022	TGF-β1/Smad3 signaling pathway ↓
Hu et al. 2022	1. CX3CL1/RAF/MEK/ERK signaling pathway ↓
	2. Inhibiting tubular cell epithelial-mesenchymal transition
Yu et al. 2022	1. Regulating AKT1/GSK3β signaling pathway
	2. Inhibiting epithelial-mesenchymal transition
Tang et al. 2022	Regulating macrophage polarization
Li et al. 2023a	1. ALDH2 ↑

	2. Inhibiting epithelial-mesenchymal transition
	3. Inhibiting G2/M arrest
Li et al. 2023b	AMPK/eNOS signaling pathway ↑
Arda et al. 2024	1. Antioxidant
	2. Anti-inflammation
Qing et al. 2024	NF-κB/NLRP3 signaling pathway ↓
Xu et al. 2025	TPL2/CTGF signaling pathway ↓
Su et al. 2025	1. HIF-1α signaling pathway ↓
	2. Regulating macrophage polarization
He et al. 2025	1. cAMP/PKA signaling pathway ↓
	2. Anti-ferroptosis

AKT: protein kinase B; ALDH2: aldehyde dehydrogenase 2; AMPK: AMP-activated protein kinase; cAMP: cyclic adenosine monophosphate; CTGF: connective tissue growth factor; CX3CL1: C-X3-C motif chemokine ligand 1; eNOS: endothelial nitric oxide synthase; ERK: extracellular signal-regulated kinase; GSK3β: glycogen synthase kinase-3β; HIF-1α: hypoxia-inducible factor-1alpha; HO-1: heme oxygenase-1; Keap1: kelch-like ECH-associated protein 1; MEK: mitogen-activated protein kinase; My D88: myeloid differentiation factor 88; NF-κB: nuclear factor Kb; NLRP3: nod-like receptor protein 3; NRF2: nuclear factor E2-related factor 2; PKA: protein kinase A; SIRT1: sirtuin 1; TGF-β: transforming growth factor-beta; TLR: toll-like receptor; TPL2: tumor progression locus 2

Supplementary Figure S1. Linear dose-response analysis charts on (A) Scr, (B) BUN, (C) TGF-β1.

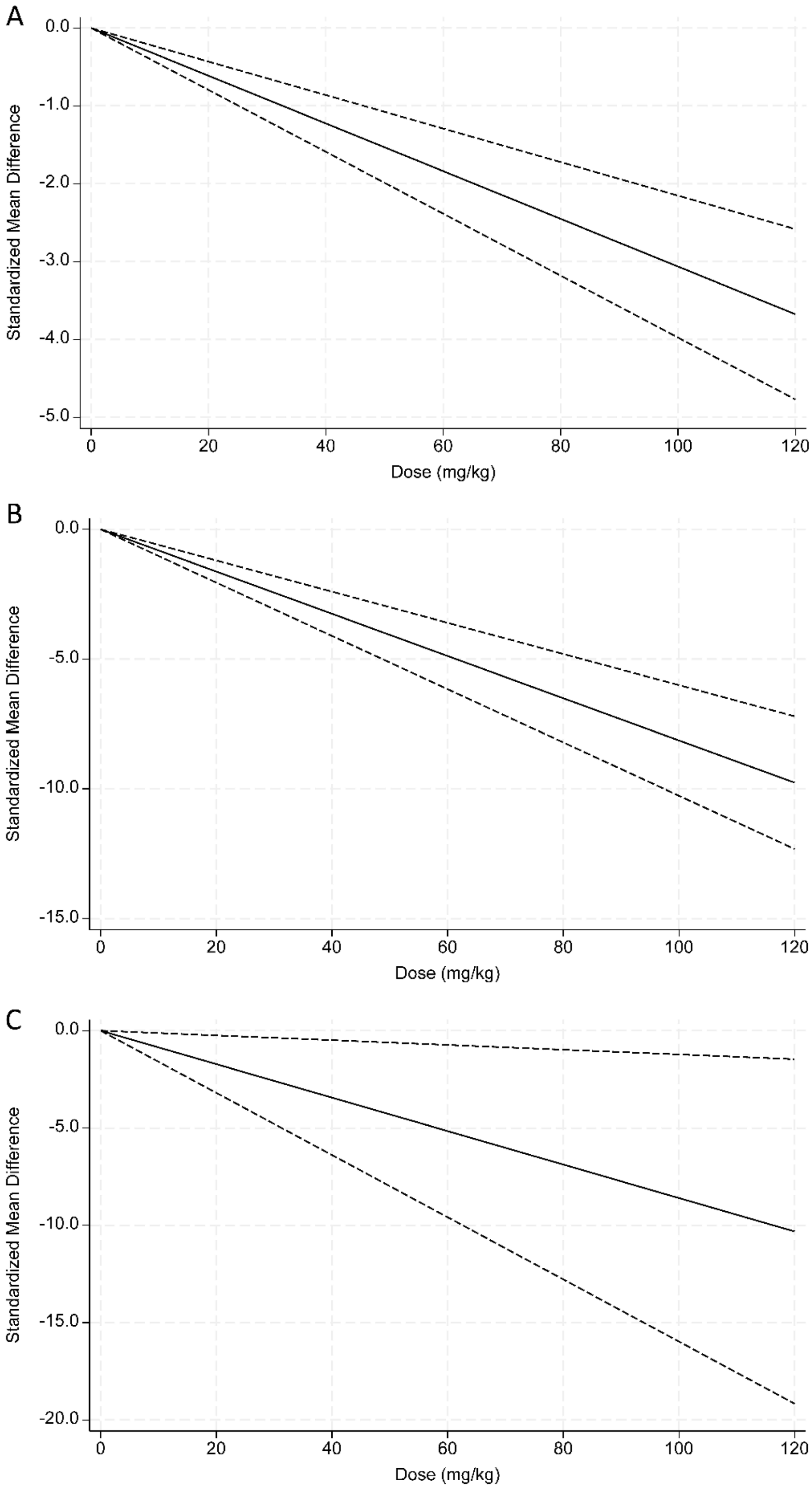

Supplementary Figure S2. Non-linear dose-response analysis charts on (A) proteinuria, (B) fibronectin, (C) α-SMA.

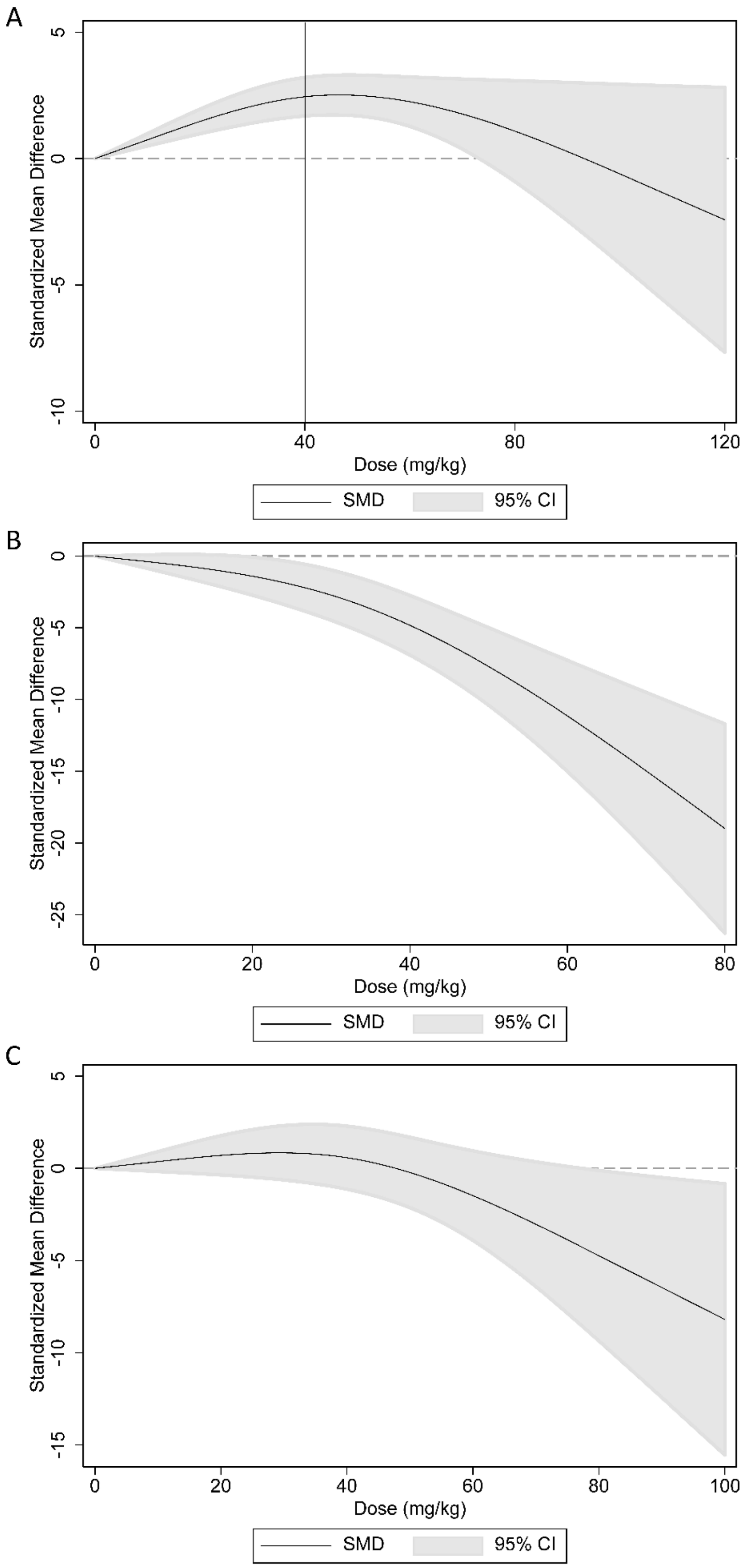

Supplementary Figure S3. Forest plot: effect of AS-IV on blood glucose.

	Experimental			Control				Std. Mean Difference
Study or Subgroup	Mean	SD	Total	Mean	SD	Total	Weight	IV, Random, 95% CI
Chen et al. 2016	22.19	4.79	10	27.48	7.78	10	12.3%	-0.78 [-1.70, 0.13]
Fan et al. 2017	20	2.2	8	33.6	3.7	11	10.9%	-4.10 [-5.82, -2.37]
Hu et al. 2022	30.52	4.63	30	30.97	3.07	10	12.5%	-0.10 [-0.82, 0.61]
Li et al. 2023b	14.28	1.52	9	24.8	3.38	9	11.0%	-3.82 [-5.51, -2.13]
Mao et al. 2019	225.86	20.33	6	315.85	12.17	6	9.0%	-4.96 [-7.63, -2.29]
Song et al. 2018	32.66	6.24	6	30.63	3.63	6	11.9%	0.37 [-0.78, 1.51]
Su et al. 2025	51.2	4.8	20	97.2	4.8	10	9.0%	-9.32 [-11.97, -6.68]
Wang et al. 2017	17.23	1.56	10	26.81	2.85	10	11.1%	-3.99 [-5.63, -2.36]
Wang et al. 2019a	5.26	0.25	36	6.59	1.02	12	12.4%	-2.40 [-3.23, -1.58]
**Total (95% CI)**			**135**			**84**	**100.0%**	**-2.96 [-4.40, -1.51]**

Heterogeneity: $Tau^2 = 4.22$; $Chi^2 = 96.65$, $df = 8$ ($P < 0.00001$); $I^2 = 92\%$

Test for overall effect: $Z = 4.01$ ($P < 0.0001$)

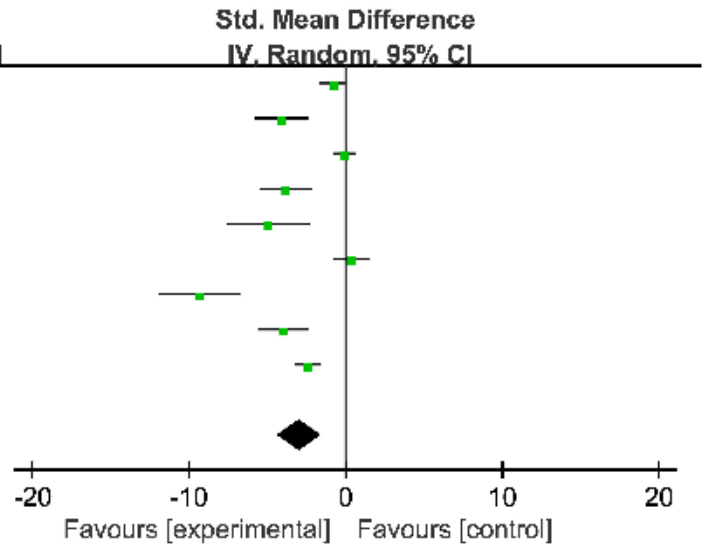

Supplement: Supplementary file 1 [file DataSheet1.pdf]
